# Physics-based modeling approaches of resistive switching devices for memory and in-memory computing applications

**DOI:** 10.1007/s10825-017-1101-9

**Published:** 2017-11-13

**Authors:** D. Ielmini, V. Milo

**Affiliations:** Dipartimento di Elettronica, Informazione e Bioingegneria and IU.NET, Politecnico di Milano, Piazza L. da Vinci 32, 20133 Milan, Italy

**Keywords:** Resistive switching memory, Memristor, Emerging memory, Nonvolatile memory, Device modeling, Transport modeling, Compact modeling, In-memory computing, Neuromorphic computing

## Abstract

The semiconductor industry is currently challenged by the emergence of Internet of Things, Big data, and deep-learning techniques to enable object recognition and inference in portable computers. These revolutions demand new technologies for memory and computation going beyond the standard CMOS-based platform. In this scenario, resistive switching memory (RRAM) is extremely promising in the frame of storage technology, memory devices, and in-memory computing circuits, such as memristive logic or neuromorphic machines. To serve as enabling technology for these new fields, however, there is still a lack of industrial tools to predict the device behavior under certain operation schemes and to allow for optimization of the device properties based on materials and stack engineering. This work provides an overview of modeling approaches for RRAM simulation, at the level of technology computer aided design and high-level compact models for circuit simulations. Finite element method modeling, kinetic Monte Carlo models, and physics-based analytical models will be reviewed. The adaptation of modeling schemes to various RRAM concepts, such as filamentary switching and interface switching, will be discussed. Finally, application cases of compact modeling to simulate simple RRAM circuits for computing will be shown.

## Introduction

Resistive switching memory (RRAM) is a 2-terminal memory device which can change its resistance in response to the application of external voltage pulses [1–4]. RRAM, also sometimes referred to as memristor [5], generally consists of a metal-insulator-metal (MIM) stack, where resistance can change as a result of a local modification of the material composition, *e*.*g*., along a conductive filamentary (CF), or within an interface layer. This marks the difference between RRAM and other resistive memory devices, such as phase change memory (PCM), where the resistance change is dictated by a different phase of the active material [6], of magnetic random-access memory (MRAM), where the resistance change results from a re-orientation of the magnetic polarization within a ferromagnetic layer [7]. RRAM offers a simple structure, CMOS compatibility, back-end of the line (BEOL) process, high speed and low power consumption. Given the large number of switching materials and their possible combination in MIM stacks [3], multilayers [8], and multi-terminal structures [9], RRAM offers an unprecedented flexibility to serve different demands of memory, storage and computing.

**Fig. 1:**
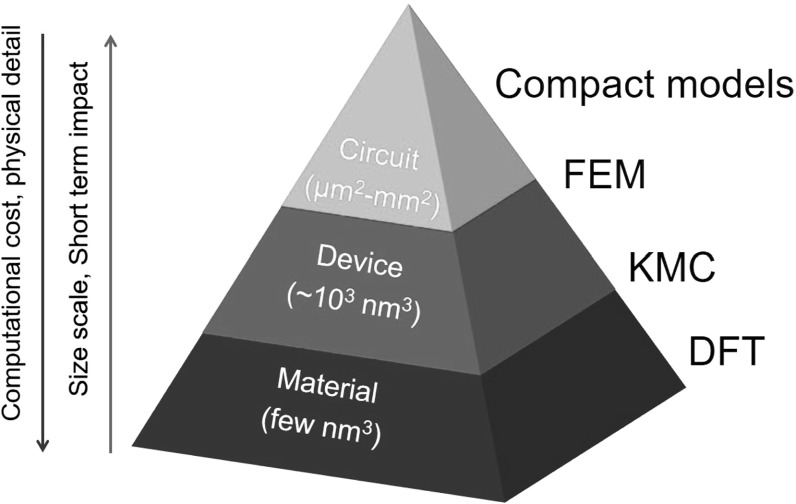
Ecosystem of physical-based models for RRAM. Ab-initio DFT models provide the materials understanding that is instrumental to simulate device operation via FEM/KMC models. The latter provide input for analytical models to simulate RRAM-based circuits. Computational cost and complexity increase with the physical detail and the smaller scale. High-level analytical models allow for a short-term impact in the form of design of applications for motivating RRAM development

**Table 1:** List of main physical modeling approaches adopted to investigate RRAM devices with a comparison as a function of scale, computational cost and capabilities

	Description	Scale	Computational cost	Capability
Atomistic	Solution of physical equations based on DFT	Few $\hbox {nm}^{3}$	High	Calculate intimate physical quantities, e.g., energy barriers for defect generation and migration, band structure, phonon structure, etc
KMC	Solution of equations for transport of heat, electrons and defects. Defects are described by individual positions	100 $\hbox {nm}^{3}$	Medium high	Calculate device characteristics such as *I*–*V* curves, *R*–*t* curves, etc. The simulation is inherently stochastic, thus average device behavior can be obtained only by several runs of the simulation tool
FEM	Solution of equations for transport of heat, electrons and defects. Defects are described by a concentration	1000 $\hbox {nm}^{3}$	Medium low	Calculate device characteristics such as *I*–*V* curves, *R*–*t* curves, etc. The model simulates the average device behavior. Variability and noise can be implemented by energy landscape stochasticity
Compact	Solution of equations describing global characteristics of the device (e.g., filament diameter, average temperature, voltage)	Several $\upmu \hbox {m}^{3}$	Low	Calculate the device characteristics by simple analytical formula, therefore enabling the simulation of large circuit including RRAM

As other novel memory concepts, the industrial development of RRAM requires the availability of accurate models for predicting the device operation, reliability and scaling. Models have been developed across the whole hierarchy of materials-level atomistic simulations, device simulation, and compact models for exploring RRAM applications in memory and computing. Figure 1 schematically shows the different modeling approaches for RRAM. Table 1 summarizes the main properties of the modeling approaches in terms of scale, computational cost, and information that can be obtained by the model, spanning from highly physical parameters (energy barrier for defect generation and migration, vacancy formation energy, etc.) to the device characteristics or circuit functions that can be available from full circuit simulations adopting compact RRAM models. Ab-initio simulation frames relying on the density functional theory (DFT) at the atomistic scale (few $\hbox {nm}^{3})$ provide the basis for a deep understanding of materials structure, ion/atom diffusion and migration mechanisms, and the impact of oxide composition on those aspects. Physically based device simulations by finite element method (FEM) models and kinetic Monte Carlo (KMC) models allow to grasp the switching mechanisms at the device scale (few tens of $\hbox {nm}^{3})$. These simulation models have the added value of providing a direct output in the form of calculated current-voltage characteristics, or calculated response to applied pulses. FEM models consist of differential equations for transport of charge carriers (electrons, holes), heat, and ionized defects (*e*.*g*., oxygen vacancies, cations) while relying on a continuous description of the microscopic physical entities, such as electric field, temperature and defect concentration. On the other hand, KMC models solve similar equations with discrete individual defects locally enhancing the conduction via, *e*.*g*., trap-assisted tunneling. As a result, KMC are inherently stochastic, as the position of defects is dictated by Monte Carlo models for generation, recombination and migration, therefore the average switching characteristics can be obtained only from several simulation runs. On the other hand, FEM naturally yields the average switching characteristics while variation characteristics can be simulated by energy landscapes of microscopic parameters, such as the energy barrier for migration. These numerical simulations allow to visualize the local dynamics of defect concentration leading to set/reset processes, thus enabling the development of compact models consisting of a simplified set of equations for macroscopic parameters, such as the average temperature, or the geometry of the conduction spot in terms of diameter of the channel, or depleted gap. Compact models are essential tools for circuit simulations, to anticipate the demonstration of storage/computing concepts thus supporting RRAM in various application frameworks to strengthen the short-term impact on the market and industry evolution. As the model scale becomes smaller, the mathematical complexity, physical detail and computational cost increase, as summarized in Table 1.

**Fig. 2:**
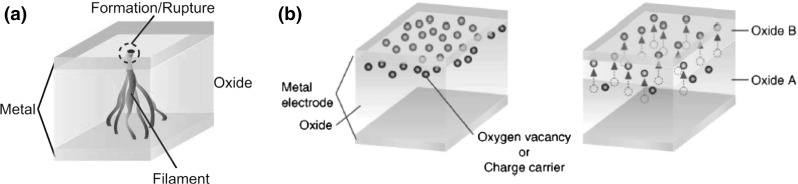
Schematic representation for **a** filamentary switching RRAM, e.g. OxRAM and CBRAM, and **b** uniform switching RRAM. Reprinted with permission from [10]. Copyright (2008) Elsevier Ltd

The purpose of this work is to provide a review of physical modeling approaches at the levels of device simulations and compact models. After briefly presenting the physical mechanisms for resistance switching, the FEM and KMC modeling approaches for RRAM will be described. Both operation and reliability modeling will be covered, as variability and noise aspects are crucial for RRAM optimization and application space. Compact models will be finally described, providing few examples of circuit applications for both memory and computing that can be efficiently studied by compact RRAM models.

## Physical mechanisms of switching

Resistive switching mechanisms can be discriminated by the type of localization of the chemical modification responsible for the change of conductance. The 2 classes of switching phenomena are shown in Fig. 2: chemical/conductance modification occurs along a filamentary path, also known as conductive filament (CF), in filamentary switching (a), whereas the change of conductance and composition occurs on an interface region in the case of uniform, or interface, switching (b).

### Filamentary switching

Filamentary switching is generally triggered by a forming operation, namely a soft breakdown operation that creates a locally degraded region with a large concentration of defects. In oxide-based RAM, also known as OxRAM, the dielectric switching layer consists of a transition metal oxide such as $\hbox {HfO}_\mathrm{x}$, $\hbox {TiO}_\mathrm{x}$ and $\hbox {TaO}_\mathrm{x}$, which is sandwiched between a top and a bottom metal electrode [1, 2, 10]. After forming, the CF shows a high concentration of metallic impurities and/or oxygen vacancies which are responsible for the low resistance state (LRS) or set state. The CF is electrically disconnected via a reset operation, which generally causes a defect depletion within a relatively limited region along the CF, thus leading to a high resistance state (HRS). The set process can recreate the CF, thus supporting filamentary switching [4].

**Fig. 3:**
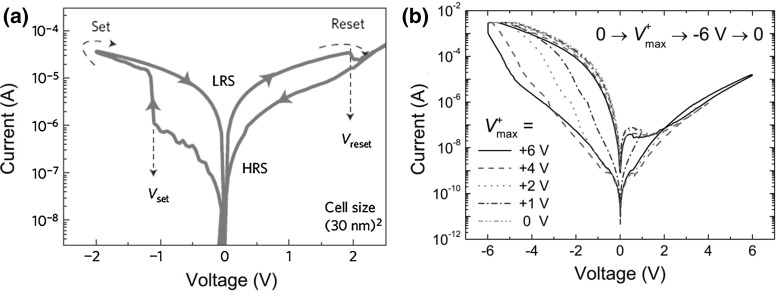
Measured *I*–*V* characteristics for **a** a $\hbox {Ta}_{2}\hbox {O}_\mathrm{5-x }\hbox {/TaO}_\mathrm{2-x}$ device featuring filamentary switching and **b** for an Al/PCMO stack with uniform switching. **a** reprinted with permission from [8]. Copyright (2011) Nature Publishing Group. **b** reprinted with permission from [24]. Copyright (2009) AIP Publishing LLC

Filamentary OxRAM can exhibit 2 switching modes depending on polarity of voltage pulses applied during set and reset operations. If both transitions occur under the positive polarity of applied voltage, resistive switching is referred to as unipolar. In unipolar OxRAM, which was originally reported in NiO [11, 12], CF formation and rupture are explained by thermally activated redox reactions [13]. In particular, the reset process leads to CF oxidation resulting in the formation of a depleted gap located at the point of CF at maximum temperature [14, 15], while the set transition involves a chemical reduction of metal oxide induced by Joule heating. In bipolar switching, instead, set and reset processes occur under opposite voltage polarities. In bipolar OxRAM, ion migration driven by electric field and accelerated by temperature is responsible for the CF connection and disruption [16]. During reset, negatively biased top electrode attracts ionized defects such as oxygen vacancies disconnecting the CF where the filament temperature is maximum. Set transition, instead, leads to a defect migration into the depleted gap region, causing the creation of a continuous CF whose size is limited by the maximum (compliance) current during the set transition, generally controlled by a transistor or resistance in series with the memory device. In particular, the same defects are migrated in one direction or the other during set/reset transitions in bipolar switching, whereas unipolar switching is assumed to require recreation of defects and their radial diffusion [17]. As a result, bipolar RRAM devices generally exhibit a higher endurance than unipolar RRAM, making bipolar switching overall more attractive for cycling intensive applications. There have been reports where the same device could show the coexistence of both unipolar and bipolar switching behaviors, such as the case of $\hbox {TiN/HfO}_{2}$ RRAM [18].

A second type of filamentary switching device is the conductive bridge RAM, also known as CBRAM [19, 20]. In CBRAM, metal impurities, typically cations supplied by Ag- or Cu-based metallic cap at the top electrode, are injected in a chalcogenide (GeSe, GeS) or oxide ($\hbox {SiO}_{2}, \hbox {Al}_{2}\hbox {O}_{3}$) electrolyte layer to create conductive paths. Set transition consists of the migration of Ag/Cu cations from the active top electrode toward the bottom electrode under a positive voltage resulting in the Ag/Cu CF formation and growth that is controlled by the compliance current. On the other hand, by applying a negative voltage to the top electrode for reset process, cations migrate in the opposite direction causing a dissolution of the metallic CF. Unipolar switching has been sometimes reported in CBRAM [21].

Despite several similarities in terms of switching and reliability between OxRAM and CBRAM devices, some differences exist. CBRAM shows a ratio between HRS and LRS resistances of about $10^{4}$ that is 2–3 orders of magnitude higher than OxRAM resistance window. The large resistance window is probably due to the higher mobility of Ag/Cu cations compared to the defects in OxRAM resulting in a larger gap and consequently in an increased HRS resistance after reset transition. As a result of the increased HRS, CBRAM devices can also operate at lower programming currents of about 10 pA [22], and feature multilevel cell operation [23].

### Uniform switching

Uniform switching, where the modification of chemical composition at the origin of the resistance change occurs within the whole device area, was evidenced in other classes of materials, such as perovskite-type oxides, *e*.*g*., PrCaMnO (PCMO) [24] and $\hbox {TaO}_\mathrm{x}\hbox {/TiO}_{2}$ bilayers [25, 26]. Uniform switching was explained as a local chemical reaction taking place at the interface between 2 separate materials. For instance, field-induced oxygen exchange can occur between a reactive top electrode and the oxide layer, *e*.*g*., between Sm top electrode and PCMO [27]. Alternatively, oxygen exchange occurs between $\hbox {TiO}_{2}$ and $\hbox {TaO}_\mathrm{x}$, where the latter serves as the barrier oxide controlling HRS/LRS resistance values [25, 26]. Figure 2b illustrates the general principles of operation for a uniform switching RRAM [10]. As a positive voltage is applied to the top electrode, oxygen ions and/or electrons drift from the bulk oxide layer toward the top electrode, thus inducing the oxidation of the electrode/oxide interface. Interface switching requires thus the use of a relatively reactive oxide, such as Al or Sm, while inert metals such as Pt do not yield significant resistance change. The resulting oxidized layer causes a resistance increase by enhancing the barrier height in a tunneling or Schottky barrier for electrons/holes injection. Application of a negative voltage results in a switching to LRS because of oxygen migration back to the bulk oxide layer. Since resistivity change occurs across the whole interface area, the HRS/LRS resistance values and the programming currents are generally proportional to the device area [28].

Filamentary and interface switching usually differ also by the shape of their *I*–*V* characteristics. Figure 3 shows the *I*–*V* characteristics for a filamentary $\hbox {Ta}_{2}\hbox {O}_\mathrm{5-x}\hbox {/TaO}_\mathrm{2-x}$ RRAM device [8] (a) and for a uniform switching RRAM with Al/PCMO structure (b) [24]. Filamentary switching is marked by an abrupt set transition, which can be explained by a sudden voltage snap back due to the sudden self-accelerated formation and growth of a CF [29]. On the other hand, uniform switching appears as smooth set/reset transition and usually shows largely asymmetric characteristics due to rectification induced by Schottky barriers or asymmetric tunneling barriers.

**Fig. 4:**
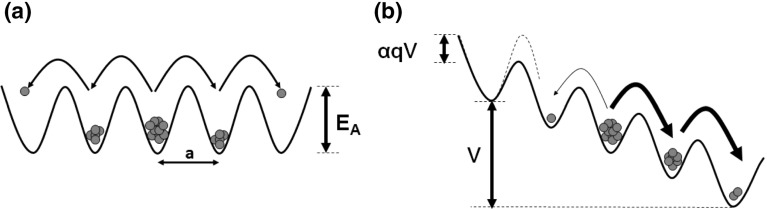
Schematic illustration of physical mechanisms controlling hopping-based migration of ionized defects in bipolar RRAM. **a** Ionic diffusion is driven by temperature and concentration gradient, while **b** ionic drift is driven by the electric field. Reprinted with permission from [30]. Copyright (2012) IEEE

## Device simulation models

RRAM switching devices have triggered strong research interest in view of their small size, easy fabrication, low current operation, and high speed. Despite these attractive features, a critical barrier for RRAM commercialization has been the lack of deep understanding and predictability of the switching characteristics. To support the development and scaling of RRAM, various types of computational models have been developed. Technology computer-aided design (TCAD) techniques enable the simulation of device conduction and switching characteristics, allowing to give a microscopic view of the switching mechanisms, to study the impact of geometry on the switching behavior, and to explore scaling of RRAM technology. Therefore, TCAD models of RRAM devices have received a strong interest from both the academia and the industry. TCAD models can be divided into 2 main classes, namely finite element method (FEM) numerical models, and kinetic Monte Carlo (KMC) models.

### Finite element method (FEM) modeling

In a FEM model, transport equations are numerically solved in 2D or 3D geometries where the volume is discretized with finite elements. While device simulations of typical Si-based devices, *e*.*g*., *p*–*n* diodes or complementary metal-oxide-semiconductor (CMOS) devices, only require the solution of carrier transport equations, RRAM simulation is much more complex as thermal and ionic effects are deeply functional to the device operation. A FEM model for RRAM thus combines the challenges of both process and device simulations of CMOS devices, since transport equations of electrons, ions and phonons must be solved in a self-consistent way.

Among these phenomena, ionic migration represents the core mechanism at the origin of the change of the chemical composition (hence resistivity) in the active material. Ion migration can be described by a hopping mechanism controlled by temperature-activated drift and a diffusion mechanism, as depicted in Fig. 4. Diffusion (Fig. 4a) is driven by concentration gradient, thus can occur even in the absence of an electric field. On the other hand, ionic drift takes place in the direction of the electric field *F* (Fig. 4b), because of the field-induced lowering of the hopping barrier [16, 30]. In the general case, the total ion-migration current density ${j}_{D}$ is given by the combination of diffusion current density ${j}_\mathrm{diff}$ and the drift current density ${j}_\mathrm{drift}$, namely:

1 $$\begin{aligned} j_D =j_\mathrm{diff} +j_\mathrm{drift} =-D\nabla n_D +\mu Fn_D, \end{aligned}$$

where ${n}_{D}$ is the ionized defect concentration, *D* is the ionic diffusion coefficient, and $\upmu$ is the ionic mobility. Note that ion diffusivity is temperature activated according to the Arrhenius law, namely:

2 $$\begin{aligned} D=D_0 e^{-\frac{E_A}{kT}}, \end{aligned}$$

where *T* is the temperature, ${E}_{A}$ is the energy barrier for hopping transport in Fig. 4a, and *k* is the Boltzmann constant. Also, ion mobility $\upmu$ depends on diffusivity according to the equation:

3 $$\begin{aligned} \mu =\frac{qD}{kT}, \end{aligned}$$

known as the Einstein relation.

The drift–diffusion ionic continuity equation $\nabla j_D =0$ must then be solved with the Poisson continuity equation for electron current, which yields *F* to enter Eq. (1), and the Fourier equation to calculate *T* entering Eq. (2). Note that this model attributes resistive switching to a pure migration of defects, without any significant generation or recombination of defects. These are assumed to be generated at forming, and remain confined in the CF region with negligible loss during the set/reset cycling.

The migration of ions within an active region, generally consisting of the CF area, results in a change of chemical composition which affects the local resistance. To describe the impact of composition on resistivity, the defects, *e*.*g*., oxygen vacancies and hafnium ions, can be considered to act as dopants in the metal oxide [30]. In fact, increasing the defect density in a metal oxide is known to affect the local density of states (DOS), by introducing states in the gap which can act as doping [31, 32]. According to this picture, the local defect concentration ${n}_{D}$ controls the electrical conductivity $\sigma$, which is assumed dependent on temperature via an Arrhenius law given by:

**Fig. 5:**
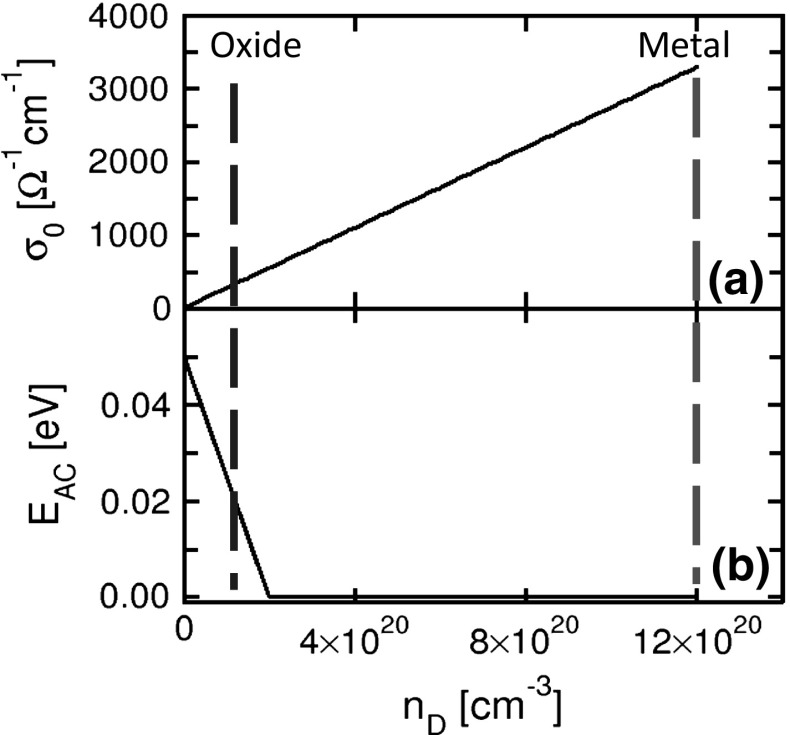
Calculated evolution of electrical conductivity parameters in Eq. (4), namely **a** the pre-exponential factor $\sigma _{0}$ and **b** the activation energy ${E}_{AC}$ at increasing of defect density ${n}_{D}$. Reprinted with permission from [30]. Copyright (2012) IEEE

**Fig. 6:**
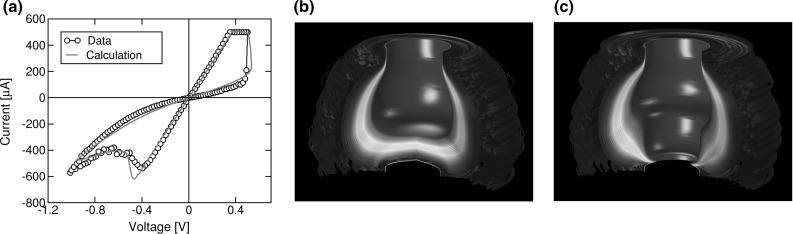
**a** Measured and calculated *I*–*V* characteristics, 3D maps of defect concentration ${n}_{D}$ describing **b** HRS and **c** LRS, respectively, obtained by a FEM model

4 $$\begin{aligned} \sigma =\sigma _0 e^{-\frac{E_{AC}}{kT}}, \end{aligned}$$

where $\sigma _{0}$ is a pre-exponential factor and ${E}_\mathrm{AC}$ is the activation energy for electrical conduction. In Eq. (4), electrical transport is assumed to obey to a thermally activated hopping mechanism, such as Poole–Frenkel, which has indeed been evidenced at relatively low conductance in RRAM devices [33]. Figure 5 shows $\sigma _{0}$ (a) and ${E}_{AC}$ (b) as a function of ${n}_{D}$ [30]. A linear increase of $\sigma _{0}$ is assumed in the calculation, to describe the transition from HRS, at low defect concentrations, to LRS at high defect concentration approaching a maximum value ${n}_{D}= 1.2\times 10^{21}~\hbox {cm}^{-3}$ at which the local conductivity becomes virtually metallic. The linear increase of $\sigma _{0}$ is consistent with both the Poole-Frenkel picture of conduction, where each carrier is thermally emitted from a localized state, and the doping theory in semiconductors, where carriers originate from the ionization of doping atoms. The activation energy ${E}_{AC}$ is assumed zero for high ${n}_{D}$, because of the doped-semiconductor or metallic-like conduction of CF in the set state, while ${E}_{AC}$ is assumed to linearly increase for decreasing ${n}_{D}$ close to zero as a result of a Poole–Frenkel-type electrical conduction in the case of disconnected filament.

Figure 6a shows measured and calculated *I*–*V* characteristics for an $\hbox {HfO}_\mathrm{x}$-based bipolar RRAM evidencing an abrupt set transition and a more gradual reset process. The latter is due to the migration of ionized defects activated by field and temperature toward the negatively biased top electrode resulting in a depleted gap along CF [16, 30]. The depletion process is seen to start close to the middle of CF, where *T* generally reaches its maximum value along the CF [30]. This physical explanation of reset process is supported by the evolution of the defect density calculated by a numerical FEM model [30], which is shown in Fig. 6b at the end of the reset transition, *i*.*e*., for the HRS. In fact, the map evidences a clear depletion region, or depleted gap, extending close to the bottom electrode. In this depleted gap, the concentration of defects is so low that the conductivity pre-factor $\sigma _{0}$ is relatively small, while the energy barrier is large according to Fig. 5, therefore resulting in a relatively large resistance in the depleted region which is at the origin of the resistance rise during the reset process. On the other hand, when a positive voltage is applied to the top electrode, ionized defects migrate in the direction of the electric field toward the bottom electrode, causing a fast increase of defect density in the depleted gap. The map of ${n}_{D}$ at the end of the set transition, namely for the LRS, in Fig. 6c shows no depleted gap and a continuous CF with low resistance.

**Fig. 7:**
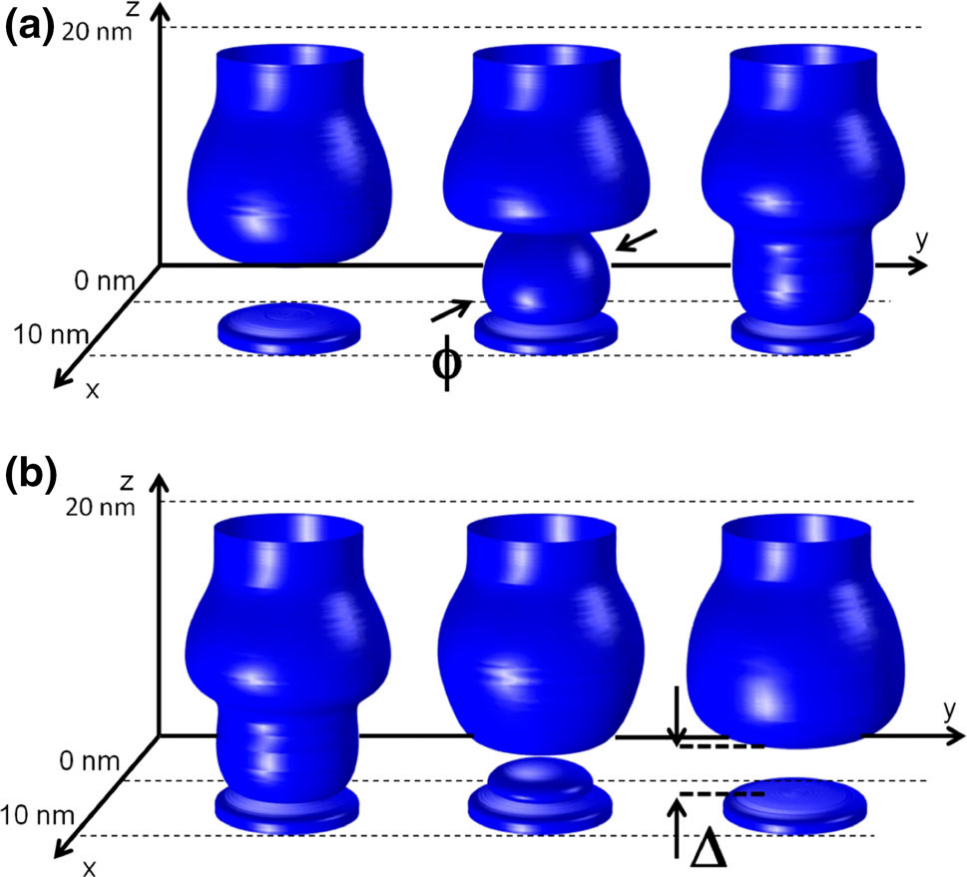
3D contour plots of the defect concentration illustrating the evolution of **a** set transition by the formation and growth of the CF and of **b** reset transition via a gradual opening of a depleted gap. Reprinted with permission from [34]. Copyright (2014) IEEE

More details about the evolution of the CF during set transition are obtained by 3D contour plots of defect density shown in Fig. 7a. From the initial HRS, the set process results in the connection of top and bottom stubs via formation of a sub CF whose diameter $\phi$ increases until reaching a maximum value limited by the compliance current. Figure 7b illustrates the evolution of CF shape during reset transition, showing the formation and the gradual opening of the depleted gap with length $\Delta$ reaching a maximum value in the HRS [30, 34].

**Fig. 8:**
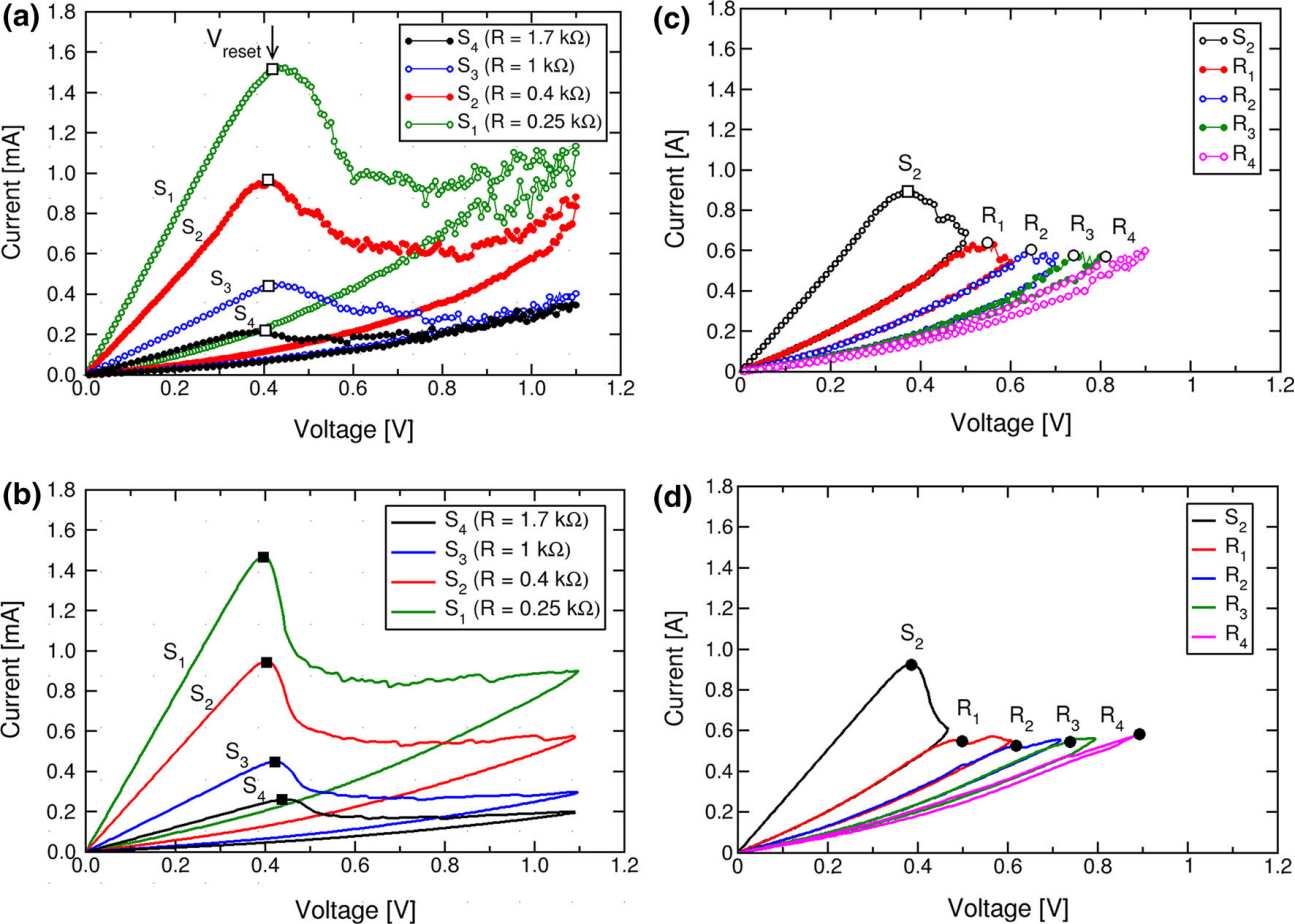
**a**, **b** Measured and calculated *I*–*V* characteristics showing reset transitions at variable initial LRS resistance (${S}_{1}-{S}_{4}$). Both measured and simulated curves evidence that ${V}_\mathrm{reset}$ does not depend on initial state. **c**, **d** Measured and calculated *I*–*V* curves for variable HRS (${R}_{1}-{R}_{4}$) obtained by voltage sweeps at increasing ${V}_\mathrm{stop}$ starting from the set state ${S}_{2}$ of resistance $R = 0.4~\hbox {k}\Omega .{ V}_\mathrm{reset}$ increases with the initial resistance of the HRS. Reprinted with permission from [35]. Copyright (2012) IEEE

Figure 8 shows the measured (a) and calculated (b) current during the reset transition as a function of the absolute value of voltage. The *I*–*V* curves are shown for various initial set states ($${S}_{1}, {S}_{2}, {S}_{3}$$ and $${S}_{4})$$ differing by their diameter $$\upphi $$, namely initial resistance increases from $${S}_{1}$$ to $${S}_{4}$$ as $$\upphi $$ decreases due to a decreasing compliance current $${I}_\mathrm{C}$$ used during the previous set transition [35]. Note that the reset voltage $${V}_\mathrm{reset}$$ is almost constant for all set states, thus the reset current linearly increases with LRS conductance 1 / *R*, or equivalently with the cross-sectional area of the CF. Note that $${I}_\mathrm{reset}\approx {I}_\mathrm{C}$$ in Fig. 8a, b, since $${V}_\mathrm{reset}$$ is almost equal to $${V}_\mathrm{C}$$, *i*.*e*., the critical voltage controlling ionic migration during set transition. Figure 8 also shows the measured (c) and calculated (d) *I*–*V* curves of reset transition for various initial states, including a set state $${S}_{2}$$ of resistance $$R = 0.4 k\Omega $$ and four reset states ($${R}_{1}$$, $${R}_{2}$$, $${R}_{3}$$, and $${R}_{4})$$ of increasing resistance. These reset states were obtained by applying consecutive reset sweeps with increasing stop voltage $${V}_\mathrm{stop}$$, namely the maximum voltage in the reset transition. As $${V}_\mathrm{stop}$$ increases, the depleted gap length $$\Delta $$ increases in the final reset state, thus *R* also gradually increases from $${R}_{1}$$ to $${R}_{4}$$. The first reset state $${R}_{1}$$ was obtained by resetting $${S}_{2}$$ with $${V}_\mathrm{stop }= 0.5$$ V. Afterward, starting from $$R_{1}$$, a second voltage sweep with $${V}_\mathrm{stop}$$ = 0.6 V is applied causing the device resistance to increase to a higher value corresponding to the reset state $${R}_{2}$$. Finally, $${R}_{3}$$ and $${R}_{4}$$ are obtained by the application of further consecutive sweeps at increasing $${V}_\mathrm{stop }$$ resulting in a further increase of R. Note that $${V}_\mathrm{reset}$$, defined as the first voltage evidencing an increase of *R*, increases with the initial resistance of the reset state in both the experimental data and the calculations, which is in contrast with the behavior of $${V}_\mathrm{reset}$$ observed for set states in Fig. 8a, b.

**Fig. 9:**
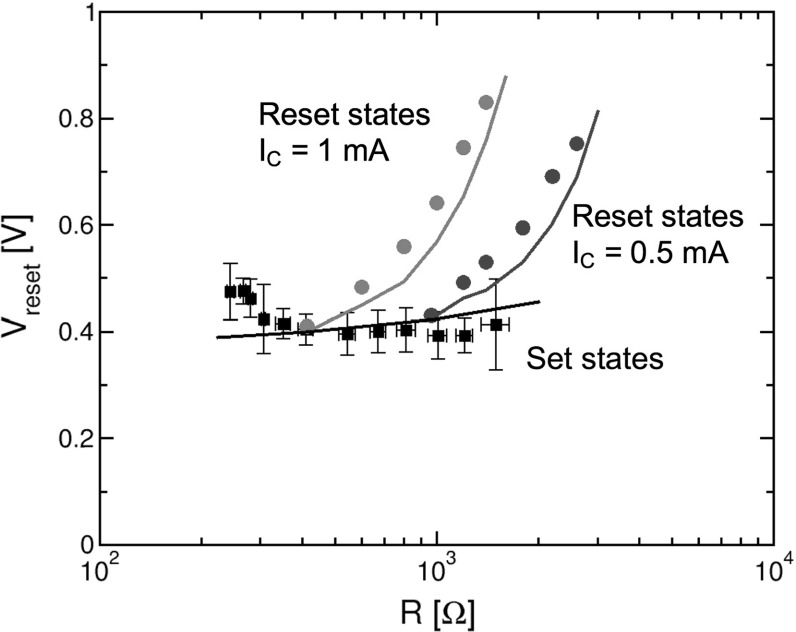
Measured and simulated ${V}_\mathrm{reset}$ as a function of *R* for variable set states, differing by ${I}_\mathrm{C}$ in the set transition, and variable reset states, differing by ${V}_\mathrm{stop}$ in the reset transition. Reset states resulting from set states obtained at two different values of ${I}_\mathrm{C}$ (0.5 and 1 mA) are compared in the figure. Note that ${V}_\mathrm{reset}$ is almost constant for set states, while it increases with *R* for reset states. Reprinted with permission from [35]. Copyright (2012) IEEE

The different behavior of ${V}_\mathrm{reset}$ is further summarized in Fig. 9, collecting the measured and calculated ${V}_\mathrm{reset}$ for variable set and reset states. Set states are achieved at variable ${I}_\mathrm{C}$ while reset states are obtained at variable ${V}_\mathrm{stop}$ starting from 2 initial set states with ${I}_\mathrm{C} = 1$ mA and ${I}_\mathrm{C} = 0.5$ mA, respectively. In the case of the set states, ${V}_\mathrm{reset}$ remains essentially constant at 0.4 V since the maximum electric field and maximum temperature in the CF are not affected by any change in CF diameter and cross section [33]. On the other hand, reset states with increasing *R* show an increasing ${V}_\mathrm{reset}$, as a result of the increasing length of the depleted gap. In fact, the electric field is strongly localized at the depleted gap, and the longer is the depleted region, the smaller is the remaining field across the conductive region of the CF, where *F* drives ionic migration at the origin of the reset transition. As a result, to activate ion migration in reset states, ${V}_\mathrm{reset}$ must increase according to the gap extension.

**Fig. 10:**
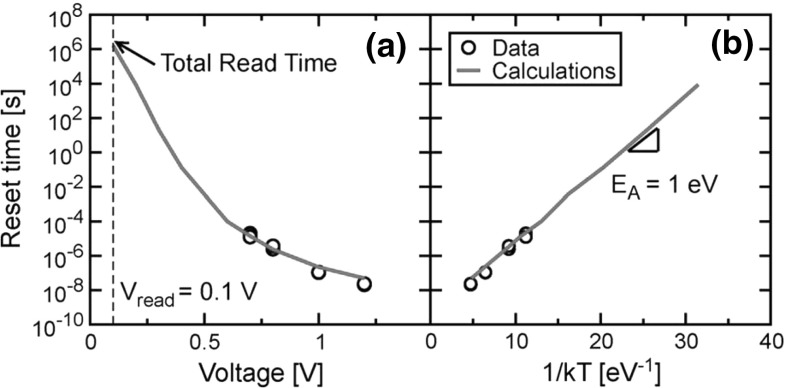
Measured and calculated evolution of reset time as a function of **a** pulse amplitude and **b** 1/kT, where *T* indicates the maximum temperature in the CF calculated by the numerical model. Reprinted with permission from [30]. Copyright (2012) IEEE

In addition to static DC characteristics as in Figs. 6 and 8, the numerical drift–diffusion model can provide accurate prediction of AC-type measurement results, such as ${V}_\mathrm{reset}$ under variable sweep rate, or reset time at constant voltage. Figure 10 shows the measured and calculated reset time defined as the time to observe an increase of resistance by 60 % with respect to the initial value during the reset transition at constant voltage [30, 35]. The reset time in Fig. 10a shows a highly non-linear dependence on the absolute value of the applied voltage. This can be explained by the Arrhenius dependence of diffusion kinetics in Eq. (2), where the local *T* is induced by Joule heating, thus increases approximately with the square of the applied voltage [16]. To support this explanation, Fig. 10b shows the reset time as a function of 1/kT, where *T* was evaluated from the model as the maximum temperature along the CF at the reset transition. Data and calculations show a clear exponential dependence, thus evidencing the Arrhenius dependence and supporting the crucial role of temperature in accelerating ion migration and reset transition. The FEM model thus shows a full capability to predict device behavior under both basic lab-type experiment, such as quasi-static *I*–*V* curves, and more application-driven explorations of device speed, thus satisfying the need for industrial TCAD-type simulations.

**Fig. 11:**
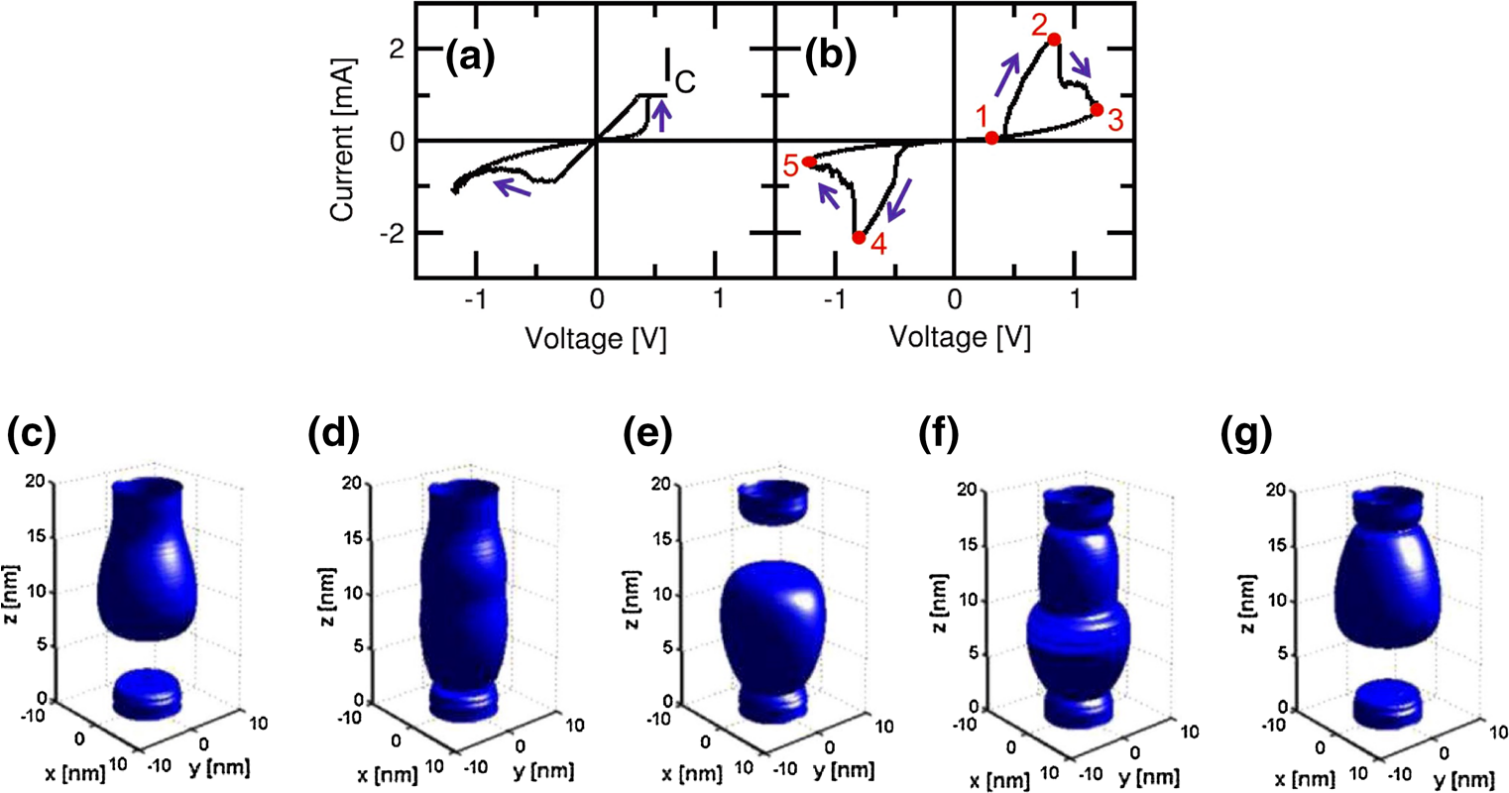
**a** Measured *I*–*V* curve with compliance current ${I}_\mathrm{C} = 1$ mA and **b** without current limitation, resulting in a CS, for the same bipolar $\hbox {HfO}_\mathrm{x}$ RRAM. An applied positive voltage induces a set transition (from state 1 to state 2) followed by a reset transition (from state 2 to state 3), finally resulting in the positive HRS, or PHRS. Starting from PHRS, an applied negative voltage induces a set transition (from state 3 to state 4) followed by a reset transition (from state 4 to state 5), finally resulting in the negative HRS, or NHRS. **c–g** Contour plots of ${n}_{D}$ depict the CF shape modifications during CS from state 1 to state 5, respectively. Reprinted with permission from [36]. Copyright (2013) IEEE

To further support the physical picture and numerical accuracy of the FEM model, simulations were carried out also to reproduce the complementary switching (CS) phenomenon, which is shown in Fig. 11 [36]. In bipolar switching (*I*–*V* curve in Fig. 11a), the amount of defect displacement along the CF is usually controlled by limiting the maximum current by an external ${I}_\mathrm{C}$, which thus limits the resistance to a value $R = {V}_\mathrm{C}/{I}_\mathrm{C}$, where ${V}_\mathrm{C}$ is the minimum voltage inducing ionic migration in the time scale of the experiment [16]. The external current limitation is suppressed in CS, thus resulting in the *I*–*V* curves shown in Fig. 11b for an $\hbox {HfO}_\mathrm{x}$ RRAM device.

Starting from an initial reset state (state 1 in Fig. 11c), an applied positive voltage in Fig. 11b causes the device to undergo a set transition where the maximum current (about 2 mA) exceeds the compliance current of 1 mA, which was initially used to set the device in the previous bipolar switching operation in Fig. 11a. This can be understood by the unlimited ionic displacement which causes CF growth above the usual limits of the bipolar switching. However, there is a self-limitation mechanism of the CF size which is enforced by the maximum availability of defects in the reservoir at the top electrode. In fact, it should be noted once again that bipolar switching is explained by ionic migration where no new defects are generally generated, since the latter phenomenon would require a much larger energy compared with simple defect migration. After the maximum current state (state 2 in Fig. 11d), the current decreases at increasing applied voltage leading to a reset transition. Reset process can be understood by ion migration from the top to the bottom electrode resulting in accumulation of defects at the bottom electrode side and opening of a depleted gap at the top electrode side. This is indicated by contour plot of ${n}_{D}$ for state 3 in Fig. 11e, with a strong accumulation of defects at the bottom electrode. State 3 is also referred to as positive HRS (PHRS), as it is obtained at the end of the positive voltage sweep [36]. From PHRS, the application of a negative voltage sweep with no current compliance limitation induces a set transition leading to state 4 (Fig. 11f), whose maximum size is again limited by defect availability in the reservoir. Finally, the state 5 (Fig. 11g) is obtained by the migration of defects toward the top electrode side leading to a negative HRS (NHRS), which is similar to the initial state 1. The 3D contour plots of ${n}_{D}$ indicate that PHRS (Fig. 11e) and NHRS (Fig. 11g) differ by their opposite orientations, as the defect accumulation is at the top electrode side for NHRS, and at the bottom electrode side for PHRS. Calculated *I*–*V* curves by the FEM model show qualitative agreement with experimental results, thus further supporting the physical basis of the model, in particular the absence of significant generation/recombination effects during bipolar/complementary switching [36].

**Fig. 12:**
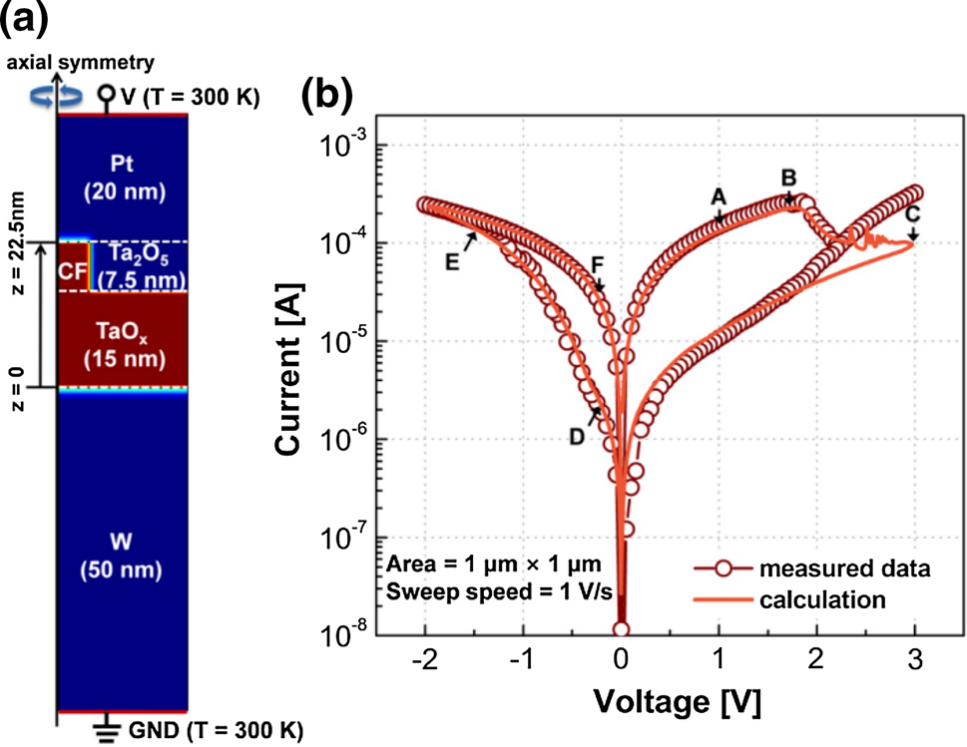
**a** Cross section of a $\hbox {Pt/Ta}_{2}\hbox {O}_{5}\hbox {/TaO}_\mathrm{x}$/W RRAM device and **b** a measured *I*–*V* characteristic with the corresponding calculation obtained by a FEM model. Reprinted with permission from [37]. Copyright (2013) Nature Publishing Group

**Fig. 13:**
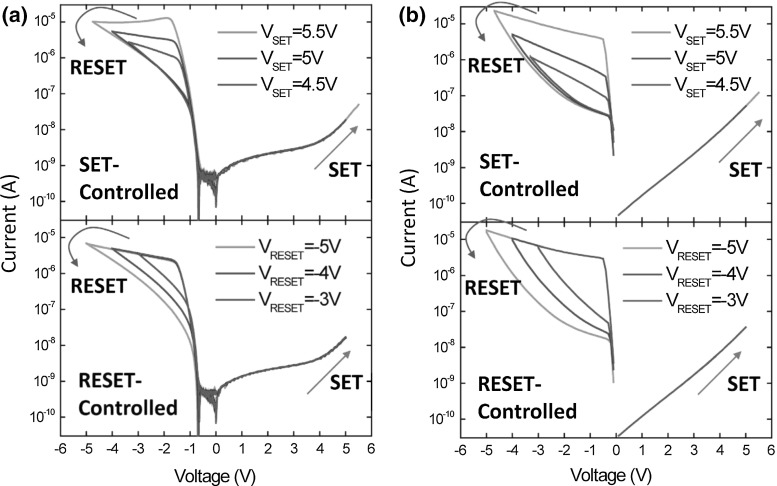
**a** Measured and **b** calculated *I*–*V* characteristics of a uniform switching $\hbox {TaO}_\mathrm{x}\hbox {/TiO}_{2}$ RRAM showing multiple resistance states achieved for variable ${V}_\mathrm{set}$ (top) and variable ${V}_\mathrm{reset}$ (bottom), respectively [25]

The same FEM scheme was adopted by other simulation works for calculating bipolar RRAM characteristics. Models were applied to various materials and device structures, *e*.*g*., the $\hbox {Pt/Ta}_{2}\hbox {O}_{5}\hbox {/TaO}_\mathrm{x}$/W [37] and $\hbox {Pd/Ta}_{2}\hbox {O}_{5}\hbox {/TaO}_\mathrm{x}$/Pd [38] bi-layered structures. Following [30], the FEM numerical model [37] describes set and reset transitions in terms of migration of positively ionized oxygen vacancies accelerated by electric field and Joule heating via a self-consistent solution of partial differential equations for ion, electron, and heat transport. Figure 12a shows a cross section of the simulated RRAM where the Pt and W electrodes are assumed as ideal heat sinks at room temperature [37]. Also, $\hbox {TaO}_\mathrm{x}$ layer serves as reservoir of oxygen vacancies for the formation of the CF with a radius of about 10 nm after forming operation. Figure 12b shows the experimental and simulated *I*–*V* characteristics for $\hbox {TaO}_\mathrm{x}$-based bilayer structure during reset and set processes. Contrary to Fig. 6, set transition takes place under negative voltage applied to the top electrode, since the negative voltage attracts oxygen vacancies and Ta impurities migrating across the top $\hbox {Ta}_{2}\hbox {O}_{5}$ barrier, thus allowing to fill the depleted region and cause electrical connection between top and bottom electrode. On the other hand, the application of a positive voltage causes repulsion of positive oxygen vacancies and Ta impurities from the top electrode, causing defect depletion at the top electrode side. The calculated *I*–*V* curves show a remarkably accurate agreement with the experimental data, further supporting the FEM model as a flexible and reliable numerical approach for TCAD-based RRAM simulations. FEM models were also extended to include the generation of defects, which can be used to predict the forming characteristics to initiate the CF switching from the pristine state [37, 38].

In addition to filamentary switching, uniform switching received a good deal of interest for the development of suitable simulation models. For instance, Fig. 13 shows the measured (a) and calculated (b) *I*–*V* characteristics of a $\hbox {Ta/TaO}_\mathrm{x}\hbox {/TiO}_{2}$/Ti RRAM device displaying uniform switching [25]. The device shows a self-rectifying ratio of $10^{3}$ and was fabricated with a vertical structure, adopting highly conformal deposition of $\hbox {Ta/TaO}_\mathrm{x}\hbox {/TiO}_{2}$ stack on the side wall of a multilayer $\hbox {Ti/SiO}_{2}$ stack [25, 26]. The asymmetric characteristics in Fig. 13 were explained by a Schottky barrier where the $\hbox {TiO}_{2}$/Ti interface acts as transparent ohmic contact. As a result, as a positive voltage is applied on device, electron migration from Ti electrode is hindered by the presence of conduction band offset at $\hbox {TaO}_\mathrm{x}\hbox {/TiO}_{2}$ interface. On the other hand, as the polarity is reversed, electron flux from Ta electrode is limited by $\hbox {Ta/TaO}_\mathrm{x}$ interface acting as a Schottky barrier. Switching in this uniform-type RRAM was explained by modulation of Schottky barrier induced by charge distribution at $\hbox {Ta/TaO}_\mathrm{x}$ interface. Specifically, oxygen ions are assumed mobile under applied electric field, while oxygen vacancies as fixed donor-like dopants. The *I*–*V* characteristics of $\hbox {Ta/TaO}_\mathrm{x}\hbox {/TiO}_{2}$/Ti device in Fig. 13a show multiple set (top) and reset (bottom) states at increasing ${V}_\mathrm{set}$ and ${V}_\mathrm{reset}$, respectively, where the multiple states can be attributed to variable charge densities in the Schottky barrier.

This physical description of $\hbox {Ta/TaO}_\mathrm{x}\hbox {/TiO}_{2}$/Ti operation was verified by a 1D numerical model [26] taking into account both oxygen migration and barrier modulation. According to this model, oxygen ions migration evidences both diffusion and drift components relying on ion hopping. Consequently, both are understood as temperature-assisted mechanisms according to an Arrhenius-type law. The drift velocity of oxygen ions driven by electric field is given by

5 $$\begin{aligned} v=afe^{-\frac{E_A }{kT}}\sinh \left( {-\frac{q\gamma aF}{kT}} \right) , \end{aligned}$$

where a is the effective hopping distance, *f* is the attempt-to-escape rate, ${E}_{A}$ is activation energy of ion migration, *T* is the temperature, $\gamma$ is a fitting parameter related to field dependence and *q* is the elementary charge. On the other hand, the diffusion component is modeled by a diffusivity coefficient $D=\frac{a^{2}f}{2}e^{-\frac{E_A }{kT}}$. In addition, time dependence of oxygen ion concentration ${N}_\mathrm{o}$ is given by the continuity equation of drift–diffusion:

6 $$\begin{aligned} \frac{\partial N_0 }{\partial t}=D\frac{\partial ^{2}N_0 }{\partial x^{2}}-v\frac{\partial N_0 }{\partial x}. \end{aligned}$$

Unlike oxygen ions, oxygen vacancies ${V}_\mathrm{o}$ are assumed as fixed donor-like dopants in $\hbox {TaO}_{x}$ layer whose concentration ${N}_{V}$ changes as $N_I e^{-x/x_0 }$, where ${N}_{I}$ is the vacancy density at $\hbox {Ta/TaO}_\mathrm{x}$ interface and ${x}_{0}$ is the decay constant.

The electric field *F* is obtained solving the Poisson equation

7 $$\begin{aligned} \frac{\partial F}{\partial x}=\frac{2q}{\varepsilon _r \varepsilon _0 }\left( {-N_0 +N_{V+} } \right) , \end{aligned}$$

where ${N}_{V+}$ is the density of positively ionized oxygen vacancies obtained at $F \ne 0$, $\varepsilon _\mathrm{r}$ is the relative permittivity of $\hbox {TaO}_\mathrm{x}$ or $\hbox {TiO}_{2}$ and $\varepsilon _{0}$ is the vacuum permittivity. Also, this 1D numerical model neglects the current transport in $\hbox {TiO}_{2}$ because the latter is limited by $\hbox {TaO}_\mathrm{x}$ Schottky barrier. Overall, numerical calculations in Fig. 13b show good qualitative agreement for both controlled set mode (top) and controlled reset mode (bottom) with data which support the validity of the model.

### Kinetic Monte Carlo modeling

Another approach being adopted to investigate switching characteristics of bipolar RRAM devices relies on Kinetic Monte Carlo (KMC) models. As opposed to FEM schemes, where all variables are treated as continuous, the KMC approach deals with discrete quantities, such as the number and position of defects.

**Fig. 14:**
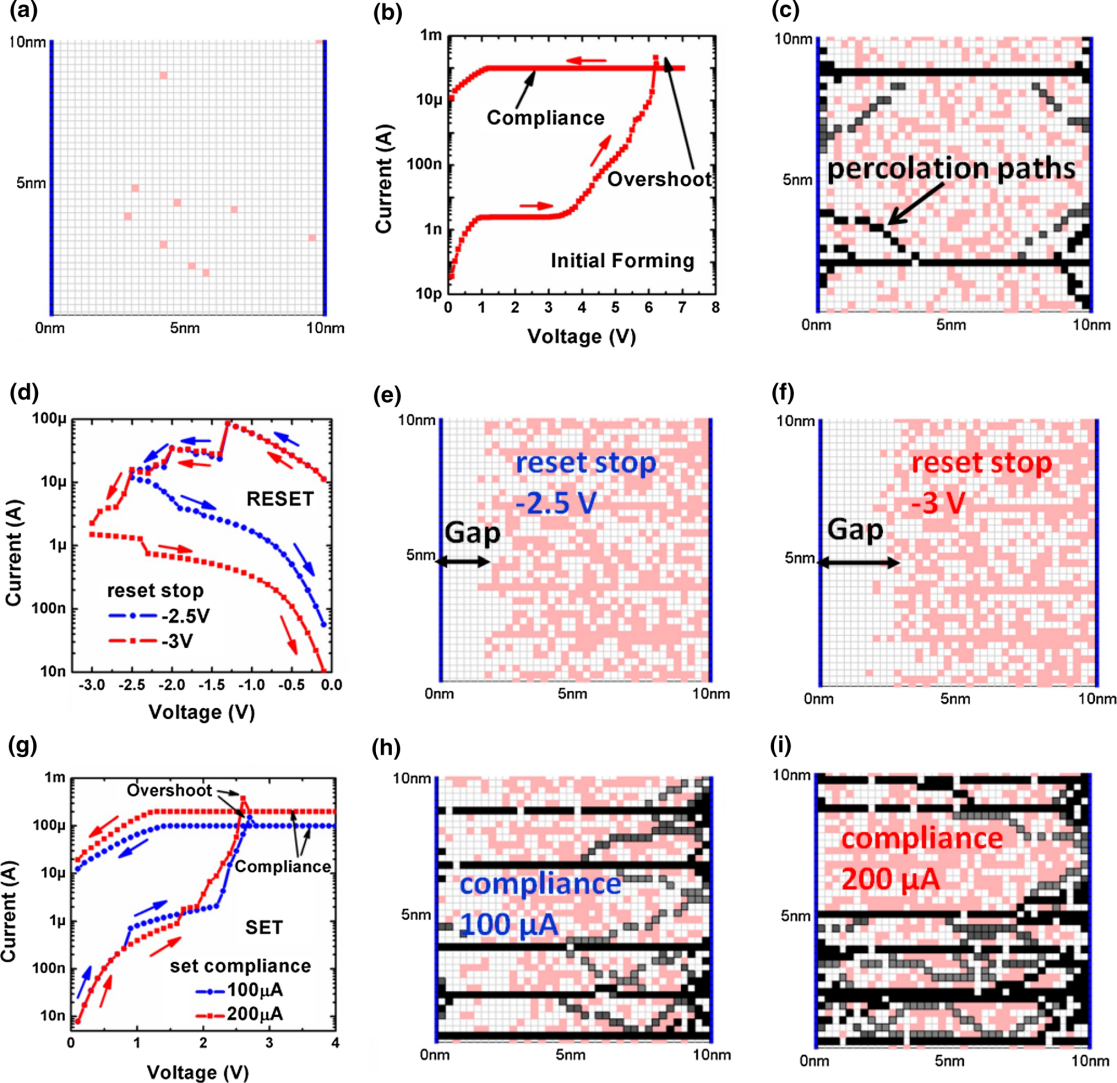
Calculated results by a KMC modeling of resistive switching for a bipolar RRAM with a 10-nm-thick $\hbox {HfO}_\mathrm{x}$ layer. **a** Starting from an initial reset state, **b** the device undergoes the forming operation leading to **c** the formation of some percolation paths. After forming, **d** RRAM can be reset by applying a negative voltage sweep whose maximum value ${V}_\mathrm{stop}$ controls the length of the gap and thus the final HRS (**e**, **f**). **g** The application of a positive voltage induces a set transition limited by ${I}_\mathrm{C}$ resulting in the formation of CF connecting two electrodes. **h**, **i** The number of CFs increases for higher ${I}_\mathrm{C}$, thus leading to lower LRS. Reprinted with permission from [39]. Copyright (2011) IEEE

The KMC numerical model presented in [39] assumes generation/recombination of oxygen vacancies as the main origin of resistance change, and trap-assisted tunneling (TAT) as the mechanism responsible for conduction in resistive switching devices. Based on TAT conduction interpretation, electrons tunnel from cathode into the closest oxide trap and from there hop through a chain of oxygen vacancies until reaching, by tunnel, the anode. Figure 14 shows calculations performed by this model for forming, reset and set processes of a 2D RRAM with an $\hbox {HfO}_\mathrm{x}$ dielectric layer of thickness 10 nm [39]. As indicated in Fig. 14a, the RRAM device initially shows a high resistance owing to random spatial configuration of few defects in the oxide layer. During forming, whose *I*–*V* curve is shown in Fig. 14b, the application of a high positive voltage on pristine device results in the formation of percolating paths because of the fast increase in oxygen vacancies driven by electric field and temperature on the basis of a positive feedback mechanism. At the end of forming, a defect configuration as the one in Fig. 14c is obtained. After forming, a reset operation is carried out on RRAM. To this purpose, Fig. 14d illustrates the *I*–*V* characteristics for two different reset transitions with ${V}_\mathrm{stop} = -2.5$ and −3 V, respectively. As confirmed by Fig. 14e, f, the reset operation with higher ${V}_\mathrm{stop}$ leads to a longer depleted gap which thus results in a higher HRS. In addition, note that depleted gaps evidence random edges, which can be due to stochastic nature of recombination process of oxygen vacancies. Also, simulated results show sequential current drops during the reset transition, which again can be attributed to random recombination events affecting the overall device resistance. Figure 14g–i shows set transitions under two different compliance currents. Set operated at higher compliance current (Fig. 14i) induces a higher amount of vacancies, thus resulting in a high number of conductive paths and thus in a lower LRS.

**Fig. 15:**
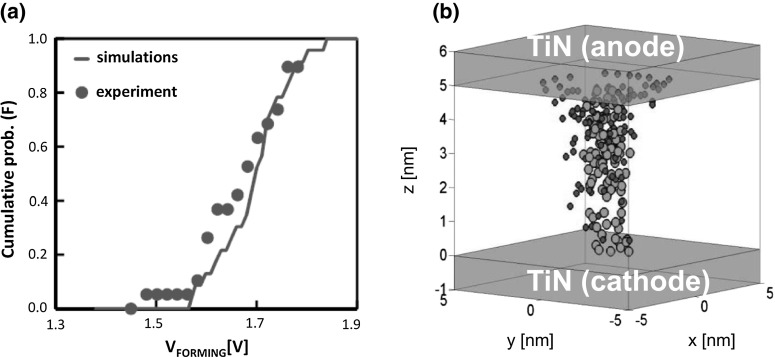
**a** Measured and calculated cumulative distributions of forming voltage for $\hbox {TiN/HfO}_{2}$/TiN RRAM at ${T}= 25$ $^{\circ }\hbox {C}$ and **b** calculated distributions of oxygen vacancies (green) and oxygen ions (red) in case of ramped voltage forming with slope 1 V/s. Reprinted with permission from [40]. Copyright (2012) IEEE (Color figure online)

The KMC model [40] was shown to allow for an accurate description of the forming process and its statistics as indicated in Fig. 15. Forming is activated by the power consumption which induces a considerable increase of temperature resulting in a high generation rate of oxygen vacancies. The oxygen vacancy generation rate G was modeled as an exponential function of temperature and electric field F given by:

8 $$\begin{aligned} G\left( {x,y,z} \right) =ve^{-\frac{E_A \left( {x,y,z} \right) -bF\left( {x,y,z} \right) }{kT\left( {x,y,z} \right) }}, \end{aligned}$$

where $\upnu$ is the Debye vibration frequency, b is the bond polarization factor, k is the Boltzmann constant, and ${E}_\mathrm{A}$ is the effective defect formation energy at $F = 0$. Oxygen vacancies result in an increase of TAT current, hence in a higher temperature leading to a positive feedback responsible for CF formation. The model allows to simulate the distribution of forming voltage in Fig. 15a, and of the spatial distribution of discrete defects, namely oxygen ions (red) and vacancies (green) as shown in Fig. 15b. It should be noted that oxygen vacancies are uniformly distributed evidencing the formation of a CF, whereas the oxygen ions move toward the top electrode interface as a result of field-assisted drift.

### Atomistic simulations

FEM numerical models provide extremely accurate results for the understanding of switching phenomena in RRAM devices. However, although the description of the fundamental mechanisms responsible for the switching process is highly accurate, as validated against several experimental results, the quantitative details of ionic drift/diffusion and the relationship between defect concentration and the thermal/electrical conductivities require more dedicated physical investigation, such as atomistic physical models, to serve as input to TCAD industrial-scale models.

**Fig. 16:**
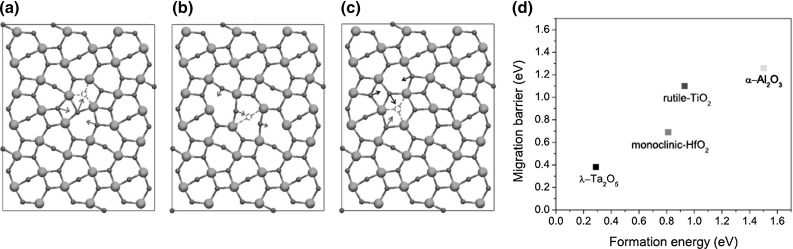
**a**–**c** Calculated oxygen vacancy migration for $\lambda$-$\hbox {Ta}_{2}\hbox {O}_{5}$ RRAM at atomic scale and **d** diagram of migration energy as a function of oxygen vacancy formation energy to compare $\lambda$-$\hbox {Ta}_{2}\hbox {O}_{5}$ to other important transition metal oxides. Reprinted with permission from [41]. Copyright (2016) AIP Publishing LLC

**Fig. 17:**
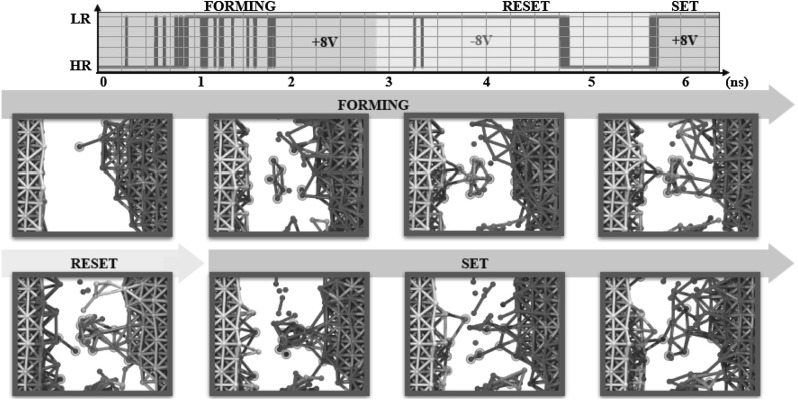
Atomistic pictorial representation of forming, reset and set transitions in ultra-scaled $\hbox {Cu/a-SiO}_{2}$ CBRAM cell. The application of a positive voltage (indicated by blue color) at Cu-based active electrode induces the dissolution of Cu ions into the $\hbox {a-SiO}_{2}$ layer forming small clusters leading to stable conductive filaments. On the other hand, as the voltage is reversed, the inactive electrode becomes positively charged causing the filament rupture at its side. Reprinted with permission from [42]. Copyright (2015) Nature Publishing Group (Color figure online)

Figure 16a–c illustrates calculated results of an atomistic model showing the oxygen vacancy migration in $\uplambda$ phase $\hbox {Ta}_{2}\hbox {O}_{5}$-based RRAM [41]. Because of adaptive crystal structure of this oxide layer, the oxygen vacancies diffuse through energy barriers inducing a rearrangement of neighboring atoms. The migration process starts with an in plane coordinated displacement of atoms leading a 3f vacancy to reach a 2f vacancy (a). Afterward, the 2f vacancy migrates toward a 3f vacancy (b) and finally, from 3f oxygen site, either the sequence 3f-2f-3f is repeated again or the vacancy directly reaches the 2f site (c) [41]. Figure 16d shows calculated migration barrier energy as a function of the oxygen vacancy formation energy for various transition metal oxides and note that $\uplambda -\hbox {Ta}_{2}\hbox {O}_{5}$ evidences the lowest energies compared with monoclinic$\hbox {-HfO}_{2}$, rutile$\hbox {-TiO}_{2}$, and $\upalpha \hbox {-Al}_{2}\hbox {O}_{3}$. This result, obtained starting from first principles, supports $\uplambda \hbox {-Ta}_{2}\hbox {O}_{5}$ RRAM as an extremely attractive solution for very fast, low power RRAM devices. A similar atomistic approach was also used to study in depth the switching kinetics of Cu/amorphous $\hbox {SiO}_{2}$ CBRAM cells at technological miniaturization limit corresponding to a cross section of $7\times 7 \,\hbox {nm}^{2}$ and an oxide thickness of 1–4 nm, namely at scaling limit [42]. To this purpose, Fig. 17 shows a time evolution of connection and disruption of a CF between two electrodes at atomic level. The atomistic simulations performed on ultra-scaled devices evidence that the application of a positive forming voltage induces Cu ions at the active electrode to become positively charged. As a result, these Cu ions tend to dissolve into the electrolyte causing the formation of small metallic aggregates. In addition, the electric field drives these Cu ions toward the inactive electrode resulting in the formation of continuous conductive bridges whose stability is enhanced by applied voltage.

After forming, the cell can be reset by the application of a negative voltage, which positively charges the filament ions close to the inactive electrode. Consequently, these Cu ions dissolve into the electrolyte causing a partial rupture of the CF, namely only at inactive electrode side. Finally, changing the polarity of applied voltage, the active electrode assumes a positive charge easily dissolving into the electrolyte and thus inducing the formation of conductive bridge paths.

## Compact modeling of RRAM

While TCAD device simulations provide high detail at the relevant scale of MIM stack and RRAM device, they are not suitable for large-scale simulations of circuits. For instance, a memory array might contain from kbit up to several Gbit of memory devices, thus requiring analytical models with an adjustable degree of simplification depending on the circuit scale. A key feature of such analytical models is the capability to incorporate device switching variability of switching voltages, *e*.*g*., ${V}_\mathrm{set}$ and ${V}_\mathrm{reset}$, and resistance values. These analytical models are best suited to support functionality at the level of the circuit of a system, thus providing a strong demonstration and motivation for RRAM usage in several memory and computing scenarios.

Several compact models for RRAM and CBRAM have been presented to date [16, 29, 43–46]. These models can either predict the resistance distribution [46], or simulate the whole switching characteristics by physically based equations of CF growth/depletion [16, 29, 43–45], including statistical variability affecting the switching voltages, currents, and resistance values [34]. Variability effects can be included with a Monte Carlo approach in circuit simulations, to allow for a realistic description of statistical effects, which are a key feature to explore random number generation with RRAM [47, 48], or to predict window failure in high-density memories.

### Simplified physical picture

**Fig. 18:**
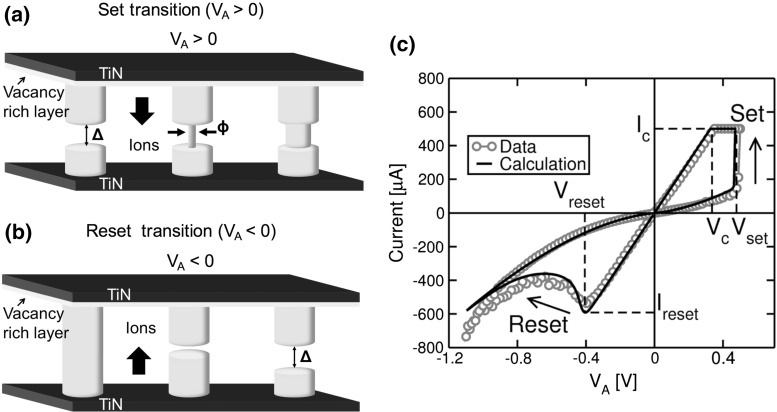
Schematic illustration of filament evolution during switching in RRAM for **a** set transition, **b** reset transition, and **c** *I*–*V* curve calculated with an analytical model, compared to experimental data for a TiN/HfO$_2$/TiN device. Reprinted with permission from [29]. Copyright (2014) IEEE

In general, the starting point for developing a compact model is to learn the switching mechanism from a detailed device simulation, such as the FEM simulation of filamentary switching shown in Fig. 7. Here, the CF shows distinctly different evolutions during set and reset processes: set transition consists of a sudden appearance of defects within the depleted gap, followed by a CF growth in terms of defect density and CF diameter within the depleted gap (Fig. 18a). On the other hand, reset transition is due to an increased length of the depleted gap (Fig. 18b). The ‘explosive’ nature of set process agrees well with the abrupt change of current in the *I*–*V* curves, compared to the more gradual transition in the reset process. The different dynamics of set and reset processes can be understood by the positive or negative feedback of electric field, temperature, and the defect distribution along the CF [29, 30]. In fact, defects during set transition migrate in response to the large electric field across the depleted gap. As defect migration starts to take place, the depleted gap length decreases, thus the local electric field increases, which further accelerates defect migration. Such positive feedback effect would result in a destructive failure of the device; however, current limitation (compliance) systems introduce an external negative feedback which allows to reduce the voltage during set transition, thus preventing destructive breakdown and enabling a detailed control of the final CF size and resistance [16, 29]. On the other hand, defect migration during reset transition is triggered by a relatively low electric field across the continuous CF. As the depleted gap starts to form, the electric field decreases in the CF regions where defects are located, thus slowing down the migration kinetics. As a result of such negative feedback effect, the voltage must be increased to further sustain the reset transition, resulting in the gradual increase of resistance.

### Simulation results

Figure 18 shows the CF evolution in filamentary-type RRAM during set (a) and reset transition (b) [29]. The CF evolution mimics the observed set/reset migration dynamics in Fig. 7, namely, set transition evolves via the growth of CF diameter $\upphi$ within the depleted gap region (a), whereas reset transition occurs by the gradual increase of the depleted gap length $\Delta$ (b). Formally, the rate equations for $\upphi$ and $\Delta$ resemble the drift/diffusion equations governing the continuous FEM modeling of RRAM [30], namely:

9 $$\begin{aligned} \frac{\hbox {d}\phi }{\hbox {d}t}=Ae^{-\frac{E_A }{kT_{inj} }} \end{aligned}$$

for set transition, where A is a pre-exponential constant, ${E}_{A}$ is a voltage dependent energy barrier for migration, and ${T}_\mathrm{inj}$ is the local temperature at the injecting CF tip, namely the one with positive potential. A similar rate equation was assumed for reset transition, namely:

10 $$\begin{aligned} \frac{\hbox {d}{\Delta }}{\hbox {d}t}=Ae^{-\frac{E_A }{kT_{inj}}}, \end{aligned}$$

where ${T}_\mathrm{inj}$ is again calculated at the positively biased, injecting CF tip [29]. These equations can be viewed as a simplified description of the CF evolution mechanism, where the CF evolves via Arrhenius-type migration dynamics controlled by an energy barrier ${E}_{A}$, and driven by the local electric field and the local temperature ${T}_\mathrm{inj}$. Figure 18c shows the measured and calculated *I*–*V* curve obtained by this model: simulation results show the same abrupt change of resistance during set transition, and a gradual change of resistance during reset transition, thus demonstrating that it correctly captures the positive/negative feedback loops controlling the microscopic CF evolution. Among the model equations, it is necessary to include (i) a shape–resistance relationship allowing to derive *R* for each value of $\phi$ and $\Delta$, and (ii) a simplified electro-thermal model allowing to estimate the local temperature ${T}_\mathrm{inj}$ based on the dissipated power V*I, and based on a detailed description of the thermal resistance controlling heat exchange across the time-varying CF and the surrounding oxide layer [29].

**Fig. 19:**
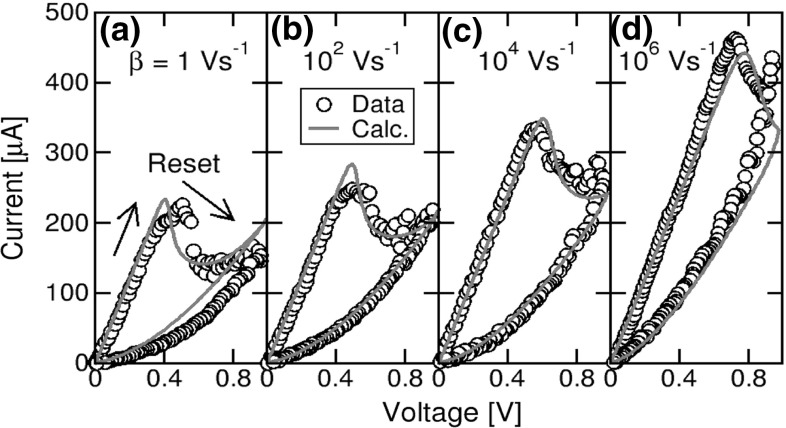
Measured and calculated *I*–*V* characteristics showing reset transition at increasing sweep rate, namely **a** $\beta = 1 \hbox {Vs}^{-1}$, **b** $\beta = 10^{2}\hbox { Vs }^{-1}$, **c** $\beta = 10^{4}$ $\hbox {Vs}^{-1}$, and **d** $\beta = 10^{6}\hbox { Vs}^{-1}$. Reprinted with permission from [29]. Copyright (2014) IEEE

In the simulation results of Fig. 18, a migration energy barrier ${E}_{A} = 1.2$ eV was assumed, thus similar to the values derived from time-dependent analysis of switching by numerical simulations [30], and similar to independent ab-initio studies of diffusion barriers in amorphous $\hbox {HfO}_{2}$ [49]. To better support the feasibility of Eqs. (9) and (10) combined with this value of ${E}_{A}$, Fig. 19 shows the measured and calculated *I*–*V* curves describing the reset transition at variable rate of the applied voltage sweep [29]. As the sweep rate $\beta = \hbox {d}V/\hbox {d}t$ was increased from 1 $\hbox {Vs}^{-1}$ to $10^{6}\hbox { Vs}^{-1}$, the reset voltage and corresponding reset current increased by about a factor 2, although the initial LRS resistance was kept constant. This is due to the time-dependent reset dynamics, where a higher local ${T}_\mathrm{inj}$, hence a higher ${V}_\mathrm{reset}$, is needed to trigger ionic migration within a shorter time according to the Arrhenius law in Eqs. (9) and (10). The analytical simulations in Fig. 19 agree very well with the experimental data, supporting the accuracy of the rate equations and of the energy barrier ${E}_{A}$ assumed in the calculations of resistance switching in $\hbox {TiN/HfO}_{2}$/TiN. Note that a different material and/or stack would lead to different values of *A* and ${E}_{A}$ in the equations; thus, this compact model requires careful adjustment to describe a specific RRAM technology.

**Fig. 20:**
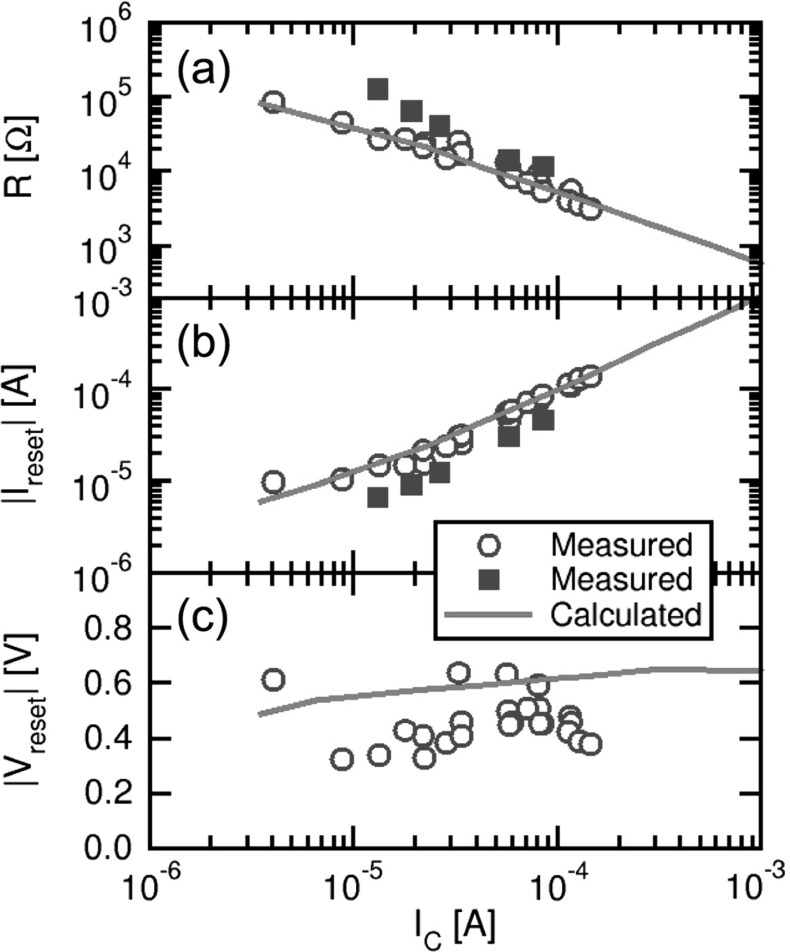
**a** Measured and calculated average LRS resistance R, **b** reset current ${I}_\mathrm{reset}$ and **c** reset voltage ${V}_\mathrm{reset}$, as a function of the compliance current ${I}_\mathrm{C}$. Data were collected for integrated one-transistor/one-resistor (1T1R) structures allowing control of the LRS in the range 10–100 ${k}\Omega$ for ${I}_\mathrm{C}$ in the range 10–100 $\upmu \hbox {A}$. Calculations agree very well with experimental data, supporting multilevel cell control of LRS resistance and low power operation of RRAM. Reprinted with permission from [34]. Copyright (2014) IEEE

The model also accounts for the dependence on current compliance ${I}_\mathrm{C}$ via the LRS resistance. Figure 20 shows the measured and calculated resistance *R* (a), reset current ${I}_\mathrm{reset}$ (b) and reset voltage ${V}_\mathrm{reset}$ (c), as a function of ${I}_\mathrm{C}$. These experimental results were collected for integrated one-transistor/one-resistor (1T1R) structures, where the small parasitic capacitance allowed for a tight control of the maximum current during set transition close to ${I}_\mathrm{C}$ and without significant overshoots [50]. As ${I}_\mathrm{C}$ decreases, LRS increases as a result of the reduced maximum CF size reached within the experimental time, which was about 1 s in the DC experiments of Fig. 20. In fact, a relatively small ${I}_\mathrm{C}$ causes a negative-feedback-induced voltage snap back to occur at relatively low current, thus forcing the final resistance to a relatively high value $R = {V}_\mathrm{C}{/I}_\mathrm{C}$, where ${V}_\mathrm{C}$ is a characteristic voltage capable of inducing ionic migration on experimental time scale [16, 29]. Analysis of data in the figure indicates ${V}_\mathrm{C}$ = 0.5 V for these experimental devices, in agreement with other RRAM device technologies including both unipolar and bipolar switching RRAMs [16]. The reset current increases with ${I}_\mathrm{C}$ as a result of the decreasing *R* and of the constant reset voltage ${V}_\mathrm{reset}$ (c). The latter is almost equal to ${V}_\mathrm{C}$, thus suggesting a symmetric behavior of ionic migration with respect to voltage polarity. Two device types differing in $\hbox {HfO}_{2}$ thickness and deposition recipe are compared in the figure [34, 51], however indicating only minor deviations. In particular, the value of ${V}_\mathrm{C}$ was shown to depend only slightly on the device material/stack and geometry parameters, such as the thickness of the oxide layer, or the length of the CF [16]. This can be explained by the analytical formula for the maximum temperature along the CF, given by:

11 $$\begin{aligned} T=T_{0} +\frac{R_\mathrm{th} }{R}V^{2}=T_0 +\frac{V^{2}}{8\rho k_\mathrm{th}}, \end{aligned}$$

where ${T}_{0}$ is the room temperature, ${R}_\mathrm{th}$/*R* is the ratio between thermal and electric resistances of the CF, V is the voltage drop across the CF, $\rho$ is the electrical resistivity and ${k}_\mathrm{th}$ is the thermal conductivity of the CF materials. The equation indicates that the local temperature does not depend on CF thickness, but is solely controlled by applied voltage since ${R}_\mathrm{th}/R$ is approximately constant. The balancing effect of thermal/electrical resistances can be explained as follows: as the thickness increases, the power dissipation ${P}={V}^{2}{/R}$ within the CF decreases, while the corresponding temperature along the CF increases. As a result, the same voltage ${V}_\mathrm{C}$ is needed to achieve the critical temperature needed to induce migration within the time scale of the experiment [16].

**Fig. 21:**
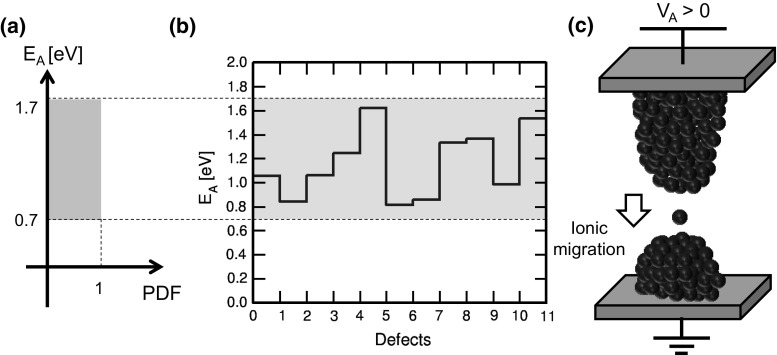
Schematic illustration of the switching variability model, based on discrete defect migration. **a** A uniform distribution of energy barriers is assumed, and **b** a random value of ${E}_{A}$ is attributed to any individual migration event during set/reset processes, corresponding to **c** an individual defect, or defect cluster. Reprinted with permission from [34]. Copyright (2014) IEEE

### Variability simulations

A key aspect of RRAM operation and its simulation for circuit applications is the statistical variability of set/reset processes. In fact, one of the purposes of circuit simulations is the prediction of the impact of device mismatch or cycle-to-cycle variations on the operation of a certain macro, such as a memory array, or a neuromorphic network, or a Boolean stateful logic circuit. In specific cases, such as a random number generator (RNG), variability is the key for circuit functionality, therefore circuit simulations inherently require that RRAM compact models feature the possibility to predict variability effects. On the other hand, numerical tools, such as the FEM or KMC models, are unfeasible for circuit simulations due to excessive computational cost, since to predict the impact of variability, one should perform Monte Carlo circuit simulations with several repeated cycles, *e*.*g*., 1 million simulations to account for a 6-sigma error rate.

To account for stochastic switching within RRAM compact simulations, one should consider that variability originates from the random environment and migration paths affecting ionic migration during set and reset processes [34]. Such randomness is captured by KMC models at the level of physical TCAD device simulation and can be introduced in FEM simulation approaches by energy landscape description of the migration barrier [34]. In analytical calculations, randomness can be introduced according to a Monte Carlo approach, where the migration barrier ${E}_{A}$ is assumed to belong to a uniform distribution as shown in Fig. 21a. Each defect is then attributed an ${E}_\mathrm{A}$ value (Fig. 21b), randomly extracted from the distribution of Fig. 21a, and controlling the defect migration process, *e*.*g*., during set transition (Fig. 21c). This approach well describes random migration effects in a simplified scheme for fast simulations within an analytical model.

**Fig. 22:**
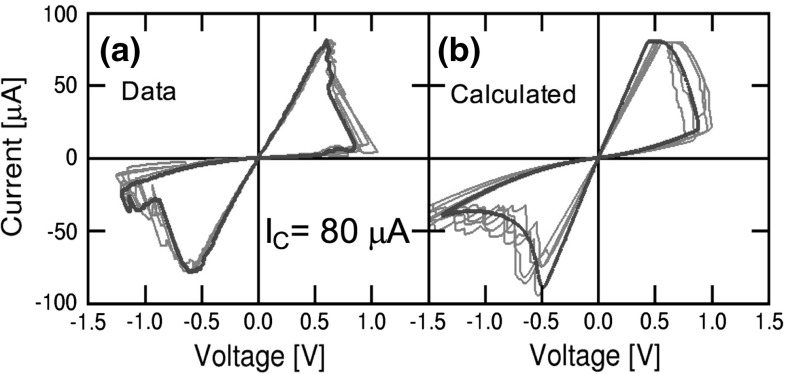
**a** Measured and **b** calculated *I*–*V* characteristics, under various measurements or Monte Carlo calculation cycles. Reprinted with permission from [34]. Copyright (2014) IEEE

Figure 22 shows the measured (a) and calculated (b) *I*–*V* characteristics evidencing stochastic switching phenomena. In the measurements, the same 1T1R device was subjected to several repeated set/reset *I*–*V* experiments for ${I}_\mathrm{C}$ = 80 $\upmu \hbox {A}$, resulting in statistical variation of all switching parameters, including ${V}_\mathrm{set}$, ${V}_\mathrm{reset}$, ${I}_\mathrm{reset}$, and resistance of LRS and HRS. In both the data and calculations, the voltage drop across the transistor was subtracted from the overall *I*–*V* curve, for best representation of the LRS resistance variability.

Calculations according to the discrete defect model in Fig. 21 show individual steps along both the set and reset transitions, which correspond to individual defects (or defect clusters) contributing to CF growth during set process, or gap depletion during reset process. Depending on the ${E}_{A}$ values of individual migration events, different values of resistance, ${V}_\mathrm{set}$ and ${V}_\mathrm{reset}$ are simulated. The simulations compare individual Monte Carlo runs with the average calculated behavior according to the standard compact model of Sect. 4.2, which evidences that this variability model well describes the picture of stochastic variations around an average ‘ideal’ switching characteristic.

**Fig. 23:**
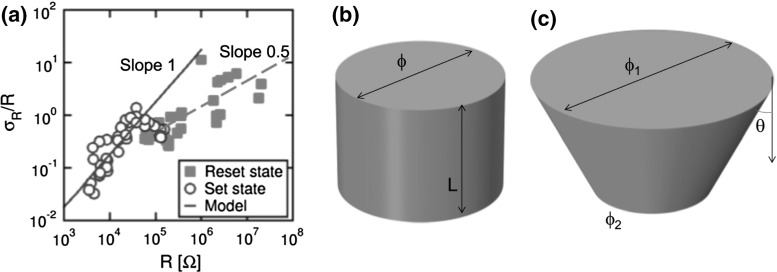
**a** Measured $\sigma _\mathrm{R}/R$ as a function of *R* for LRS and HRS and schematic illustration of a CF with **b** ideal cylindrical shape and **c** distorted conical shape [4]

Quantitative evaluations of the relative variation of LRS resistance, which can be expressed by the standard deviation of resistance $\sigma _\mathrm{R}$ divided by the average R, show that $\sigma _\mathrm{R}/R$ decreases for increasing ${I}_\mathrm{C}$, or equivalently increases for increasing *R*. This is due to the increasing number of defects involved in the set process, which results in an increased averaging among individual stochastic events, and in a consequently smaller variability. Theoretical investigations [51] and simulation results [34] indicate that the number variation causes $\sigma _\mathrm{R}/R$ to increase as ${R}^{0.5}$, due to the Poisson distribution of defect number in the CF. As shown in Fig. 23a, this model agrees well with the HRS slope [4]; however, it does not account for the observed behavior of LRS, indicating a much higher slope and linear increase of $\sigma _\mathrm{R}/R$ with *R*.

The R-dependent variability of LRS can be well described by the shape variation of the CF [4]. If we assume an ideal cylindrical shape of the CF (Fig. 23b) with a diameter $\phi$ and length L, stochastic variation in the migration paths might eventually result in non-ideal CF conformations, such as the conical shape in Fig. 23c, where the cylindrical shape is distorted by an angle $\uptheta$, although the volume is not significantly affected and may be assumed to coincide with the ideal cylindrical one. According to a simplified analysis, the cone-CF resistance can be written as:

12 $$\begin{aligned} {{R}^\prime }= \frac{4\rho L}{\pi \phi _{1}\phi _{2}}\approx \frac{4\rho L}{\pi (\phi +L\theta )(\phi -L\theta )} \end{aligned}$$

where $\upphi _{1}$ and $\phi _{2}$ are the 2 CF diameters at the top and bottom electrodes, respectively. A comparison with the ideal cylindrical resistance $R=\frac{4\rho L}{\pi \phi ^{2}}$ yields a variation:

13 $$\begin{aligned} \sigma _R =\frac{4\rho L}{\pi \upphi ^{2}}\left( {\frac{1}{1-\left( {\frac{L\theta }{\upphi }} \right) ^{2}}-1} \right) \approx R\left( {\frac{L\theta }{\upphi }} \right) ^{2}. \end{aligned}$$

From Eq. (12), one can derive a linear proportionality for the relative standard deviation, namely $\sigma _\mathrm{R}{/R}\sim { R }$ as shown by LRS data in Fig. 23a [4]. These results suggest that *R* variability is controlled by number variation in HRS, and shape variation in LRS.

**Fig. 24:**
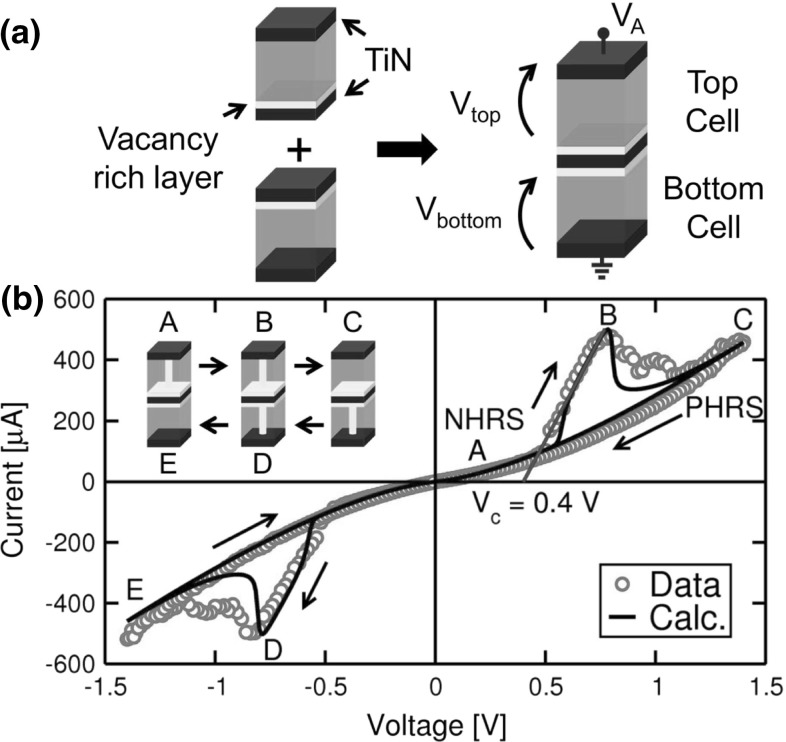
**a** Structure of the complementary resistive switch and **b** corresponding measured and calculated *I*–*V* curve. Reprinted with permission from [29]. Copyright (2014) IEEE

**Fig. 25:**
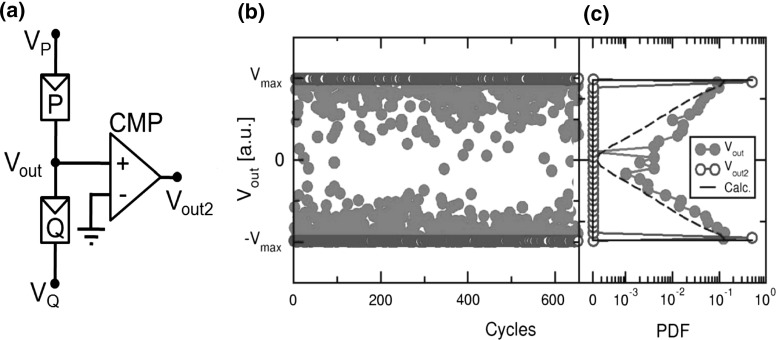
**a** Circuit schematic for the generation of random numbers, **b** measured stream of random output voltages ${V}_\mathrm{out}$ and ${V}_\mathrm{out2}$, and **c** their distributions compared to calculated results from stochastic Monte Carlo circuit simulations. Reprinted with permission from [48]. Copyright (2016) IEEE

### Circuit simulations

The major strength of the analytical model is its capability to handle even a hard circuit complexity and yield simulation in a relatively short time. To highlight the compact model capability, Fig. 24 illustrates the case of a simple circuit, namely the complementary resistive switch (CRS) combining 2 RRAM devices in anti-serial arrangement [29, 52, 53]. Figure 24a shows the connection of the 2 RRAM devices by their active electrode, the one with the oxygen vacancy layer to serve as reservoir during set transition. After cell connection, this common electrode becomes floating and is never electrically accessed, except for monitoring the electrostatic potential [29]. Figure 24b shows the measured and calculated *I*–*V* curves, including a schematic of the evolution of the states in the 2 cells (top and bottom cells) during the positive voltage sweep. The CRS was obtained by connecting 2 RRAM devices consisting of a stack of $\hbox {TiN/HfO}_{2}$/TiN which were electrically formed [29]. Initially (point A), the devices are in a negative high resistance state (NHRS), namely a state which has been obtained applying a high negative voltage resulting in reset of the bottom cell, and set of the top cell. The application of a moderate positive voltage around 0.5 V results in set transition in the bottom cell, by injection of oxygen vacancies from the intermediate electrode toward the bottom electrode. In fact, most of the voltage drops across the bottom cell which is in a HRS. Starting from the set event in the bottom cell, the voltage is equally divided between the 2 cells which are both in the LRS. A further increase of voltage (point B) causes reset of the top cell, by retraction of the CF from the top electrode toward the intermediate electrode. After the reset transition is initiated, the increasing voltage allows to complete the CF retraction in the top cell as a result of the gradual reset transition in the $\hbox {TiN/HfO}_{2}$/TiN device. At the end of the positive sweep (point C), the device is found in a positive high resistance state (PHRS), where the top cell is in HRS and the bottom cell is in LRS. The reduction of the positive voltage toward zero leaves the cell in this state, which externally shows an overall high resistance. The application of a negative voltage sweep shows a similar transition, including set transition of the top cell, followed by reset of the bottom cell and resulting in a NHRS state [29, 52, 53].

**Fig. 26:**
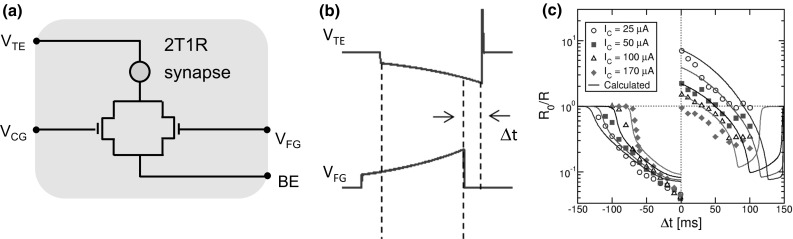
**a** Circuit schematic for the 2T1R synapse, **b** calculated waveforms of pre-synaptic (top) and post-synaptic (bottom) spikes inducing STDP, and **c** the corresponding STDP characteristics at variable initial LRS state for increasing ${I}_\mathrm{C}$ [57]. **a** Reprinted with permission from [56]. Copyright (2016) IEEE

Simulation results of CRS in Fig. 24b show a highly accurate description of the *I*–*V* curve for both positive and negative polarities, including the presence of a non-zero intercept of the LRS characteristic at zero current: the intercept is in fact given by ${V}_\mathrm{C}$, namely about 0.4 V in this device technology. This non-zero intercept is due to the fact that, while the bottom cell undergoes set transition at increasing current ${I}_\mathrm{C}$, the voltage drop across it is constantly equal to ${V}_\mathrm{C}$, namely the minimum voltage needed to sustain ion migration in the device. On the other hand, the voltage across the top cell increases linearly with ${I}_\mathrm{C}$ and is much less than ${V}_\mathrm{C}$, since the top cell is already in LRS and no ionic migration needs to be sustained. Thus, the overall voltage across the device is $V(I) = {V}_\mathrm{bottom} + {V}_\mathrm{top} = {V}_\mathrm{C} + {R}_\mathrm{LRS}*I$, which leads to the non-zero intercept in Fig. 24b.

Note that both NHRS and PHRS have a high resistance; thus, the CRS may in principle show no LRS during read. This allows for the design of CRS crossbar arrays with no selectors, since there is no significant leakage current in half-selected HRS cells. In reality, due to the relatively low resistance window in oxide-based RRAM, the HRS leakage is quite remarkable and prevents the application of CRS in select-free crossbar arrays. Higher scale circuit simulations with the analytical model allow to assess the feasibility of a CRS-based selector-free crossbar array [29].

Figure 25 shows stochastic Monte Carlo simulations of a random number generator (RNG) obtained by assembling 2 RRAM devices in the relatively small circuit shown in Fig. 25a. Here, 2 RRAM devices *P* and *Q* are arranged in a serial connection between voltage supplies ${V}_\mathrm{P}$ and ${V}_\mathrm{Q}$, respectively. The intermediate voltage ${V}_\mathrm{out}$ is connected either to a third generator, or to the input of a comparator (CMP), which in turn yields a second output voltage ${V}_\mathrm{out2}$. Both cells are initially prepared in LRS, by externally applying suitable positive voltages ${V}_\mathrm{P}-{V}_\mathrm{out}$ and ${V}_\mathrm{out}-{V}_\mathrm{Q}$ in a preliminary preparation phase [48]. The second phase is the random reset operation, where symmetric voltages ${V}_\mathrm{P}$ and ${V}_\mathrm{Q}$ are applied with ${V}_\mathrm{P} < { V}_\mathrm{Q}$, thus resulting in an overall negative voltage across both *P* and *Q*. As a result, reset transition is initiated in one of the two devices, either *P* or *Q*. Assuming that reset operation starts in P, the negative voltage across *P* increases because of its increasing resistance, whereas the voltage across *Q* decreases because of a voltage divider effect. This results in a positive feedback which causes even further reset in *P* and prevents any possible reset transition in *Q*. Due to the statistical distribution of the reset voltage, the probability that reset transition starts in *P* is 50%, equal to the probability that reset transition starts in *Q*, which makes this simple circuit a useful RNG. After the random reset phase, devices are independently set to prepare for a new RNG cycle.

Figure 25b shows the measured ${V}_\mathrm{out}$, under the application of a relatively low positive voltage ${V}_\mathrm{P}-{V}_\mathrm{Q}$ for probing the final states of *P* and *Q* during a sequence of 650 RNG cycles. The output voltage shows a random distribution of positive and negative values, corresponding to reset transitions having occurred in the bottom cell *Q* or top cell *P*. The figure also shows the second output voltage ${V}_\mathrm{out2}$ which regenerates ${V}_\mathrm{out}$ by passing it into a comparator. Figure 25c shows the measured distribution of ${V}_\mathrm{out}$, and the corresponding distribution calculated by the analytical model including Monte Carlo description of stochastic reset transition. By assuming a realistic distribution of ${V}_\mathrm{reset}$, the simulation of the circuit in Fig. 25a could yield accurate prediction of the RNG results, thus supporting the high accuracy and feasibility of variability-aware circuit simulations for exploring new RRAM functions and applications.

While the simulation of digital circuits for RRAM is relevant for exploring CRS memory [29], RNG [48], and Boolean logic gates [54, 55], analog computing circuits may also benefit from realistic analytical simulations of RRAM. Figure 26 shows an example for an analog computing circuit block, namely a 2-transistor/1-resistor (2T1R) synapse circuit capable of spike-timing dependent plasticity (STDP) [56, 57]. In STDP, the conductance of a synapse is increased (potentiated) when the synapse received a spike from the pre-synaptic neuron first, followed by a spike received from the post-synaptic neuron. On the other hand, if the pre-synaptic spike follows the post-synaptic spike, then the synapse conductance (weight) is decreased (depressed). Since STDP is a well-known biological phenomenon [58, 59], artificial synapses capable of mimicking the same STDP behavior might allow for neural networks capable of unsupervised learning of patterns and associative memory, similar to the brain [60].

To meet this challenge, the 2T1R circuit in Fig. 26a consists of a RRAM device (1R) connected to 2 transistors in parallel configuration. The 2T1R synapse is connected to the pre-synaptic neuron by the communication gate (CG) of the left transistor and the top electrode, while it is connected to the post-synaptic neuron by the fire gate (FG) of the right transistor and the bottom electrode. Figure 26b (top) shows the pre-synaptic spike applied to the top electrode of the synapse, including a negative pulse with increasing voltage, followed by a positive spike. At the same time, a CG pulse is applied to the transistor to enable a relatively small current flow across the synapse, which reaches the post-synaptic neuron through the bottom electrode connection. After integrating all incoming spikes, the post-synaptic neuron eventually reaches the threshold for fire, *i*.*e*., for delivering the post-synaptic spike shown in Fig. 26b (bottom). The latter spike consists of an exponentially increasing positive spike applied to the FG. The overlap between the pre-synaptic spike at the top electrode and the post-synaptic spike at the bottom electrode results in STDP: for negative delay, which is the case shown in Fig. 26b, the overlap takes place during the negative part of the top electrode pulse, which thus results in a reset transition of the RRAM device in the 2T1R synapse [57]. On the other hand, a positive delay (not shown) would result in an overlap between the 2 spikes during the positive peak of the top electrode pulse, thus resulting in a set transition in the RRAM device.

Figure 26c shows measured and calculated conductance change, namely ${R}_{0}/R$, where ${R}_{0}$ is the initial resistance of the device and *R* is the final resistance of the RRAM after the application of a pair of pre- and post-synaptic spikes. The RRAM was initially prepared in various set states with increasing ${I}_\mathrm{C}$, and ${R}_{0}/R$ is reported as a function of the delay $\Delta {t}$ between pre- and post-synaptic spikes. In general, ${R}_{0}/R$ shows STDP behavior, with potentiation for $\Delta {t}> 0$ and depression for $\Delta {t} < 0$. Depression may take place at some extent also for relatively large positive $\Delta {t}$, since both negative and positive pulses are applied in that case, resulting in a competition between set and reset events. Overall, the simulation results can account for the measured STDP characteristics, including the analog variation ${R}_{0}/R$ during both set and reset transition. This is due to the carefully designed waveform of the pre- and post-synaptic spikes, where both the negative voltage of the top electrode pulse and the positive voltage of the FG exponentially increase with time, to allow for a $\Delta {t}$-controlled modulation of potentiation/depression. These results confirm the strong value of analytical compact models for simulating and designing RRAM circuits in various scenarios, including digital/analog applications, even in operation mode where stochastic variations are instrumental to achieve the expected behavior of the circuit. The development of even more accurate, universal compact models for RRAM will further boost the current exploration of RRAM-based circuits for analog/digital in-memory computing.

## Conclusions

This work provides an overview of various modeling approaches to RRAM devices and RRAM-based circuits. TCAD models, such as the FEM and KMC tools, provide physically based valuable tools for industrial development of RRAM devices with the ability to study not only operation, but also reliability and scaling. The extension of such models to novel RRAM device technologies, such as the uniform switching RRAM, is still a work in progress. On the other hand, analytical models for assisting the design of memory and in-memory computing circuits allow for fast simulation and exploration of novel applications, thus serving as valuable tools for future exploitation of RRAM functionalities in novel computing scenarios, such as digital Boolean mem-computing and brain-inspired neuromorphic computing.
